# c-FLIP as a master regulator of immune homeostasis and disease mechanisms

**DOI:** 10.1038/s41419-026-08771-5

**Published:** 2026-04-22

**Authors:** Eliana Urbini, Annalisa Adamo, Yushu Hu, Luca Giacobazzi, Silvia Dusi, Francesco De Sanctis, Carmine Carbone, Davide Gibellini, Sara Pilotto, Vincenzo Corbo, Vincenzo Bronte, Fulvia Vascotto, Stefano Ugel

**Affiliations:** 1 Immunology Section, Department of Medicine, University of Verona and Verona University Hospital Trust Verona, Verona, Italy; 2 Department of Medical and Surgical Sciences, Medical Oncology, Fondazione Policlinico Universitario “Agostino Gemelli” IRCCS, Rome, Italy; 3 Microbiology Section, Department of Diagnostic and Public Health, University of Verona and Verona University Hospital Trust, Verona, Italy; 4 Section of Innovation Biomedicine - Oncology Area, Department of Engineering for Innovation Medicine (DIMI), University of Verona and Verona University Hospital Trust, Verona, Italy; 5 Department of Engineering for Innovation Medicine, University of Verona, Verona, Italy; 6 ARC-Net Research Centre, University of Verona, Verona, Italy; 7 TRON, Translational Oncology at the University Medical Center of the Johannes Gutenberg University Mainz gGmbH, Mainz, Germany

**Keywords:** Apoptosis, Cell death and immune response, Immunoediting, Autoimmune diseases, Immunopathogenesis

## Abstract

Cellular FLICE (FADD-like IL-1β-converting enzyme)-inhibitory protein (c-FLIP) was discovered more than three decades ago and has since emerged as a multifunctional regulator of cell fate. Initially identified through its homology with viral FLIP (v-FLIP) proteins and its ability to inhibit death receptor–induced apoptosis, c-FLIP is now recognized as a pivotal molecule at the crossroads of apoptosis, necroptosis, autophagy, and inflammation. Beyond its classical anti-apoptotic role, c-FLIP modulates key signaling pathways, including nuclear factor-κB (NF-κB), mitogen-activated protein kinase (MAPK), and Wnt/β-catenin, thereby influencing immune cell activation, differentiation, and tolerance. In immune cells, c-FLIP expression determines susceptibility to death receptor signaling and fine-tunes inflammatory responses, contributing to the balance between immune activation and suppression. Aberrant c-FLIP regulation has been implicated in cancer, autoimmunity, and chronic inflammatory diseases, positioning it as both a biomarker and a potential therapeutic target. This review summarizes current understanding of c-FLIP structure, isoforms, and regulation; delineates its roles in apoptosis and non-apoptotic pathways; and discusses its critical function in orchestrating immune homeostasis and disease pathogenesis. By integrating mechanistic and translational perspectives, we highlight c-FLIP as a central hub that links cell death, immunity, and therapeutic opportunities.

## Facts

- Beyond its canonical role at the DISC, c-FLIP exerts pleiotropic functions across multiple cellular compartments (lipid rafts, nucleus, mitochondria-associated ER membranes), through mechanisms that remain only partially understood. How these spatially distinct pools of c-FLIP are coordinated and prioritized under physiological versus pathological conditions warrants systematic investigation.
- The balance among c-FLIP isoforms, cleavage fragments, and post-translationally modified forms appears to act as a finely tuned molecular rheostat controlling apoptosis, necroptosis, pyroptosis, autophagy, and inflammatory signaling. The rules governing this balance and how it is dynamically reset during immune activation, stress responses, or disease progression remain largely speculative and warrant in-depth exploration.
- c-FLIP-dependent regulation of cell death pathways and inflammatory signaling is tightly intertwined, suggesting that survival and inflammation are not parallel outputs but rather mechanistically coupled processes. The extent to which c-FLIP actively coordinates this coupling—rather than passively modulating individual pathways—represents an open and conceptually important question.
- The interaction between c-FLIP–mediated cell death control and metabolic or stress-response pathways (e.g., ER stress, ROS, nutrient sensing, epigenetic remodeling) suggests a bidirectional feedback loop that may amplify or restrain pathology. These interdependencies are incompletely defined and constitute a fertile area for future research.
- Given its dual role as both a protector of immune homeostasis and a facilitator of pathological persistence in cancer, chronic inflammation, and autoimmunity, c-FLIP exemplifies a context-dependent regulator rather than a simple on/off switch. Understanding how c-FLIP transitions from a physiological safeguard to a pathogenic driver is a critical unresolved issue with major therapeutic implications.

## Introduction

The cellular FLICE (FADD-like IL-1β–converting enzyme)–inhibitory protein, widely known as c-FLIP, was independently identified by multiple research groups in the late 1990s [1]. Early studies established its role as a molecular brake on apoptosis by competing with caspase-8 for recruitment to the death-inducing signaling complex (DISC), thereby determining whether cells live or die in response to death receptor activation [2]. Because evasion of apoptosis is one of the hallmarks of cancer, c-FLIP rapidly gained attention as a promising therapeutic target capable of modulating programmed cell death [3]. Subsequent discoveries expanded its known functions, revealing that c-FLIP not only governs apoptosis but also influences necroptosis [4], autophagy [5], and diverse signaling cascades involved in inflammation and immunity [6, 7].

Structurally, c-FLIP resembles caspase-8 but lacks proteolytic activity, enabling it to function as a regulatory adaptor rather than an executioner protease [8]. This unique configuration allows c-FLIP to integrate apoptotic and pro-survival signals in a context-dependent manner. It acts as a “molecular rheostat,” tipping the balance between cell death and survival depending on its isoform composition, expression level, and interaction partners [9]. The transcriptional regulation of the c-FLIP gene (*CFLAR*) involves multiple transcription factors, including nuclear factor-κB (NF-κB), activator protein-1 (AP-1), E2F1, and c-Myc, as well as epigenetic and post-transcriptional mechanisms mediated by histone modifiers and microRNAs. Such complex regulation allows c-FLIP to respond dynamically to inflammatory cues, cytokines, and metabolic stress. Significantly, NF-κB not only induces c-FLIP expression but is itself activated by c-FLIP cleavage fragments, establishing a feedback loop that maintains immune cell survival under stress conditions [10]. Evolutionary and structural parallels between c-FLIP and its viral homologs (v-FLIPs) further underscore its immunological significance. Many oncogenic viruses, such as Kaposi’s sarcoma–associated herpesvirus (KSHV), encode v-FLIPs that mimic c-FLIP’s anti-apoptotic activity to protect infected cells and promote latency [11–13]. These viral proteins often hijack host signaling pathways, such as NF-κB, to promote chronic inflammation and oncogenic transformation. The study of v-FLIPs has thus provided critical insight into how endogenous c-FLIP regulates host immune responses and contributes to disease pathogenesis.

Within the immune system, c-FLIP functions as a guardian of immune cell homeostasis. By blocking caspase-8–dependent apoptosis, it ensures the survival of essential immune populations, including T cells, dendritic cells (DCs), and monocytes, particularly during activation or inflammation [14–18]. Conversely, excessive c-FLIP expression can impede activation-induced cell death (AICD), allowing autoreactive or malignant lymphocytes to persist. Therefore, appropriate modulation of c-FLIP is vital to maintaining immune balance, preventing both immunodeficiency and autoimmunity [9]. In addition to its anti-apoptotic role, c-FLIP influences intracellular signaling networks that determine immune cell differentiation and effector functions. It participates in NF-κB and MAPK activation, regulates inflammasome assembly, and shapes cytokine production. These non-apoptotic functions connect c-FLIP directly to innate and adaptive immunity. In macrophages and DCs, for example, c-FLIP modulates Toll-like receptor (TLR) responses and controls the magnitude of inflammatory cytokine release [19, 20]. In T lymphocytes, c-FLIP determines survival and polarization, influencing the Th1/Th2 balance and the stability of regulatory T cells (Tregs) [9, 21, 22]. Dysregulated c-FLIP expression can therefore reshape the immune landscape, linking it to diseases such as rheumatoid arthritis, multiple sclerosis, and cancer [22–24]. Given its central role in coordinating immune responses, c-FLIP represents an attractive therapeutic target. Strategies aimed at reducing c-FLIP levels can sensitize tumor cells to apoptosis and enhance the efficacy of immunotherapy, whereas controlled upregulation may preserve immune tolerance in autoimmune conditions. Understanding the dualistic nature of c-FLIP, as both protector and perpetrator, remains a critical challenge in immunology.

This review provides an integrated overview of c-FLIP biology, from its molecular regulation and isoform diversity to its function in immune cell homeostasis and disease. We highlight mechanistic insights that connect c-FLIP to apoptosis, necroptosis, and inflammatory signaling, and we discuss its translational implications as a diagnostic and therapeutic target in immune-mediated pathologies.

### Biology of FLIP proteins

#### Discovery and viral FLIP proteins (v-FLIPs)

Between 1997 and 1998, at least seven groups independently identified c-FLIP and its viral counterparts, assigning multiple names, including Casper, CASH, FLAME, CLARP, MRIT, and I-FLICE [25–31]. These discoveries stemmed from efforts to understand how members of the TNF receptor superfamily trigger apoptosis. Wallach’s group at the Weizmann Institute described the protein CASH, which contained tandem death effector domains (DEDs) and showed both cytoprotective and cytotoxic activities through its interaction with death receptors CD95 (Fas receptor, FasR) and TNFR1 [25]. Concurrently, Alnemri’s laboratory cloned FLAME-1, a FasR/TNFR1-regulated anti-apoptotic factor [26]; and Shu and Halpin identified the 55-kDa protein Casper, which interacted with Fas-associated death domain (FADD), caspase-8, caspase-3, and Toll/IL-1 receptor–domain adaptors TRIF-1 and TRIF-2 [27]. Dixit’s group later cloned I-FLICE, a novel inhibitor of apoptosis mediated by CD95 and TNFR1 [28], while Núñez identified CLARP, a catalytically inactive caspase-8 homolog [29]. Tschopp’s group in Lausanne and both Peters’ and Krammer’s in Heidelberg subsequently unified these observations, designating the human protein c-FLIP and establishing its key regulatory role at DISC [30, 31].

**Fig. 1:**
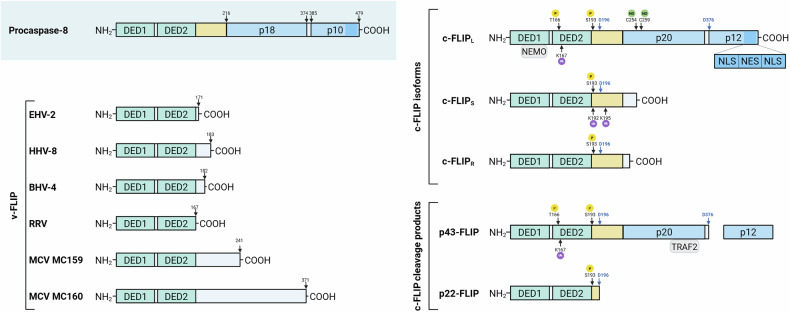
The structure of vFLIPs and the isoforms and cleavage products of c-FLIP. Viral FLICE-inhibitory proteins (v-FLIPs) have been identified in the poxvirus responsible for molluscum contagiosum (MCV MC159 and MC160 proteins), as well as in several members of the *Gammaherpesviridae* family, including bovine herpesvirus 4 (BHV-4), equine herpesvirus 2 (EHV-2), human herpesvirus 8(HHV-8), and rhesus rhadinovirus (RRV). A defining feature shared by all v-FLIPs is the presence of two death effector domains (DEDs), which are also found in the prodomains of caspase-8. In humans, c-FLIP exists in distinct spliced variants, three of which are expressed as proteins: c-FLIP long (c-FLIP_L_), c-FLIP Raji (c-FLIP_R_), and c-FLIP short (c-FLIP_S_). All three share the two DEDs and differ in their C-terminal ends. The DED1 domain has been identified as a binding site for the NF-kB modulator NEMO in all FLIP proteins. c-FLIP_L_ features additional caspase-like domains, known as p20 and p12. The c‑terminal region of c‑FLIP_L_ also contains a nuclear localization signal (NLS) and an adjacent nuclear export signal (NES), enabling nucleocytoplasmic shuttling. Caspase-mediated processing of c-FLIP_L_ at aspartate residue 376 (D376) yields the cleavage product p43-FLIP; meanwhile, caspase-cleavage at aspartate 196 (D196) produces p22-FLIP from either the long or short c-FLIP isoforms. Several post-translational modifications regulate c-FLIP proteins. Lysines 192 and 195 (K192 and K195) were identified as critical ubiquitin acceptor sites that regulate specifically the turnover of c-FLIP_S_ through the ubiquitin-proteasome system (UPS). In contrast, serine 193 (S193) serves as a phosphorylation target across all FLIP splice variants, which reduces ubiquitination and has been shown to enhance the stability of c-FLIP_S_ and c-FLIP_R_. TRAF2, one of the most well-known E3-ubiquitin ligases, interacts specifically with p43-FLIP. ROS induce phosphorylation at threonine 166 (T166) and ubiquitination at lysine 167 (K167) on c-FLIP_L_, targeting it for proteasomal degradation. S-nitrosylation of c-FLIP at cysteine residues 254 (C254) and 259 (C259) prevents its ubiquitination and proteasomal degradation.

The identification of c-FLIP closely followed the discovery of viral FLIP proteins (v-FLIPs), which provided mechanistic insight into viral immune evasion [11, 12]. Many large DNA viruses encode anti-apoptotic proteins that prevent host-cell suicide, thereby prolonging viral persistence [12]. v-FLIPs share the hallmark tandem DEDs of cellular c-FLIP and inhibit caspase-8–dependent apoptosis (Fig. 1). Representative v-FLIPs occur in poxviruses and members of the *Gammaherpesviridae* family, including bovine herpesvirus 4, equine herpesvirus 2, herpesvirus saimiri, rhesus rhadinovirus, and Kaposi’s sarcoma–associated herpesvirus 8 (KSHV/HHV-8) [13, 32–36]. The crystal structure of molluscum contagiosum v-FLIP (MC159) revealed two DEDs that mediate binding to FADD and caspase-8 [37, 38].

In KSHV, the v-FLIP K13 protein, encoded by open reading frame 71 (ORF71), exemplifies how viruses exploit FLIP-mediated signaling. v-FLIP/K13 constitutively activates the canonical and non-canonical NF-κB pathways [39–44]. Through a tumor-necrosis-factor-receptor–associated factor (TRAF)–interacting motif (PYQLT), v-FLIP/K13 binds TRAF2 and NEMO (IκB-kinase-γ), stabilizing the IKK complex and driving transcription of NF-κB target genes that prevent apoptosis and promote inflammation [43–46]. Unlike other v-FLIPs such as MC159 and E8 from equine herpesvirus 2, v-FLIP/K13 does not block FasR-induced apoptosis but instead favors chronic NF-κB activation, establishing viral latency [47–50].

Experimental mouse models confirmed the pathogenicity of v-FLIP/K13. B-cell–specific expression of v-FLIP/K13 impairs germinal center (GC) formation, immunoglobulin class switching, and affinity maturation, thereby fostering lymphoproliferation [51]. Endothelial-restricted expression provokes systemic inflammation with elevated cytokines such as interleukin-6 (IL-6), IL-10, TNF-α, and interferon-γ (IFN-γ), recapitulating the vasculopathy of Kaposi’s sarcoma [52]. Myeloid-specific v-FLIP expression drives cytokine-release syndrome (CRS) characterized by granulocyte infiltration, T-cell exhaustion, and regulatory T-cell expansion through dysregulated NF-κB/STAT3 signaling [53–55]. Collectively, v-FLIPs prevent apoptosis and sustain inflammation, allowing viruses to evade immune surveillance and contribute to oncogenesis.

### Cellular FLIP proteins (c-FLIP)

The human c-FLIP gene (*CFLAR*) resides on chromosome 2q33-q34 and spans approximately 50 kilobases [30]. Its promoter contains multiple transcription-factor–binding sites, including NF-κB, AP-1, nuclear factor of activated T cells (NFAT), E2F1, c-Myc, interferon-regulatory factor 5 (IRF5), and specificity protein 1 (SP1). Among these, NF-κB, NFAT, and SP1 serve as primary transcriptional activators, whereas c-Myc and FOXO3a act as repressors [10, 56]. Epigenetic mechanisms also regulate c-FLIP: histone deacetylase (HDAC) inhibitors, such as suberoylanilide hydroxamic acid (SAHA/vorinostat), downregulate c-FLIP transcription and sensitize tumor cells to death-receptor–induced apoptosis [57–60]. Conversely, the histone-demethylase KDM4C can enhance *CFLAR* expression by removing repressive H3K9me3 marks in a glutamine-metabolism–dependent manner [61]. At the post-transcriptional level, microRNAs (miRNAs) such as miR-708 and miR-512-3p bind to the 3′ untranslated region (3′ UTR) of c-FLIP mRNA, reducing its stability and increasing cellular susceptibility to apoptosis [62, 63]. These multilevel regulatory circuits ensure tight control of c-FLIP expression across tissues and immune contexts.

In humans, at least 13 *CFLAR* splice variants exist, but only three encode proteins: c-FLIP-long (c-FLIP_L_), c-FLIP-short (c-FLIP_S_), and c-FLIP-Raji (c-FLIP_R_) (Fig. 1) [30, 31, 64]. Alternative splicing of exon 7 determines whether translation stops early to produce c-FLIP_S_ or continues to yield c-FLIP_L_; retention of intron 6 generates c-FLIP_R_ [65–67]. All isoforms share two N-terminal DEDs that mediate interactions with FADD and procaspase-8 within the DISC, but they differ at the C-terminus. c-FLIP_L_ contains two inactive caspase-like subunits (p20 and p12) lacking catalytic residues, whereas c-FLIP_S_ has a unique 19-amino-acid tail. Caspase-mediated cleavage of c-FLIP_L_ produces the p43-FLIP and p22-FLIP fragments, which actively modulate signaling [30, 64, 68, 69]. Specifically, cleavage of c-FLIP_L_ at aspartate residue 376 (D376) located between the p12 and p20 subunits yields p43-FLIP. Meanwhile, cleavage at aspartate 196 (D196), placed between the tandem DEDs and the caspase-like domain, produces p22-FLIP from either the long or short c-FLIP isoforms. Over time, multiple nomenclature systems have been used to designate *CFLAR* splice variants. In 2005, Hainsworth et al. described two splice variants as the most widely distributed, termed cFLIP-α (MW ~ 55 kD) and cFLIP-δ isoforms (MW ranging from 25 to 28 kD), corresponding to the long and short *CFLAR* isoforms, respectively [70]. Nevertheless, the modern nomenclature consistently defines the three protein-encoding *CFLAR* isoforms as c-FLIP_L_, c-FLIP_S_, and c-FLIP_R_.

Since c-FLIP isoforms are primarily degraded via the ubiquitin-proteasome pathway, they are generally short-lived proteins. These isoforms exhibit different half-lives: approximately 3 h for c-FLIP_L_ and 30 min for c-FLIP_S_, reflecting rapid turnover via the ubiquitin-proteasome system [71, 72]. Post-translational modifications critically influence c-FLIP stability and function. Upon TNF‑α stimulation, the E3 ligase Itch ubiquitinates c-FLIP_L_, marking it for degradation, whereas the deubiquitinase USP8 counteracts this process [73, 74] [75]. Reactive oxygen and nitrogen species (ROS and RNS, respectively) also modify c-FLIP: S-nitrosylation of cysteine residues 254 and 259 prevents its ubiquitination, prolonging its anti-apoptotic activity [76]. Through these reversible modifications, cells dynamically adjust c-FLIP levels to balance survival and death signaling. Functionally, c-FLIP acts as a molecular scaffold that connects death-receptor engagement to multiple downstream pathways. By competing with procaspase-8 for binding to FADD at the DISC, c-FLIP blocks full caspase activation and apoptosis [30, 31]. Beyond apoptosis, c-FLIP_L_ binds Beclin-1 to stabilize autophagic complexes [77] and interacts with mitogen-activated protein kinase 7 (MKK7) to restrain prolonged JNK activation and reactive-oxygen-species accumulation [78, 79]. Cleavage fragments p43-FLIP and p22-FLIP activate NF-κB by engaging the IκB-kinase (IKK) complex [69, 80, 81]. These diverse interactions position c-FLIP as an essential hub that integrates apoptotic, autophagic, and inflammatory signals in a context-dependent manner.

Collectively, insights from viral v-FLIPs and their cellular homologs, c-FLIPs, highlight a conserved evolutionary strategy for controlling cell fate. Both families share structural DED modules that interfere with caspase-8 activation while recruiting adaptors to the NF-κB and MAPK pathways. In immune and epithelial cells, c-FLIP maintains survival under physiological stress, whereas dysregulation of c-FLIP contributes to tumorigenesis, chronic inflammation, and defective immune responses.

### c-FLIP in cellular processes

#### Regulation of cell death pathways

**Fig. 2:**
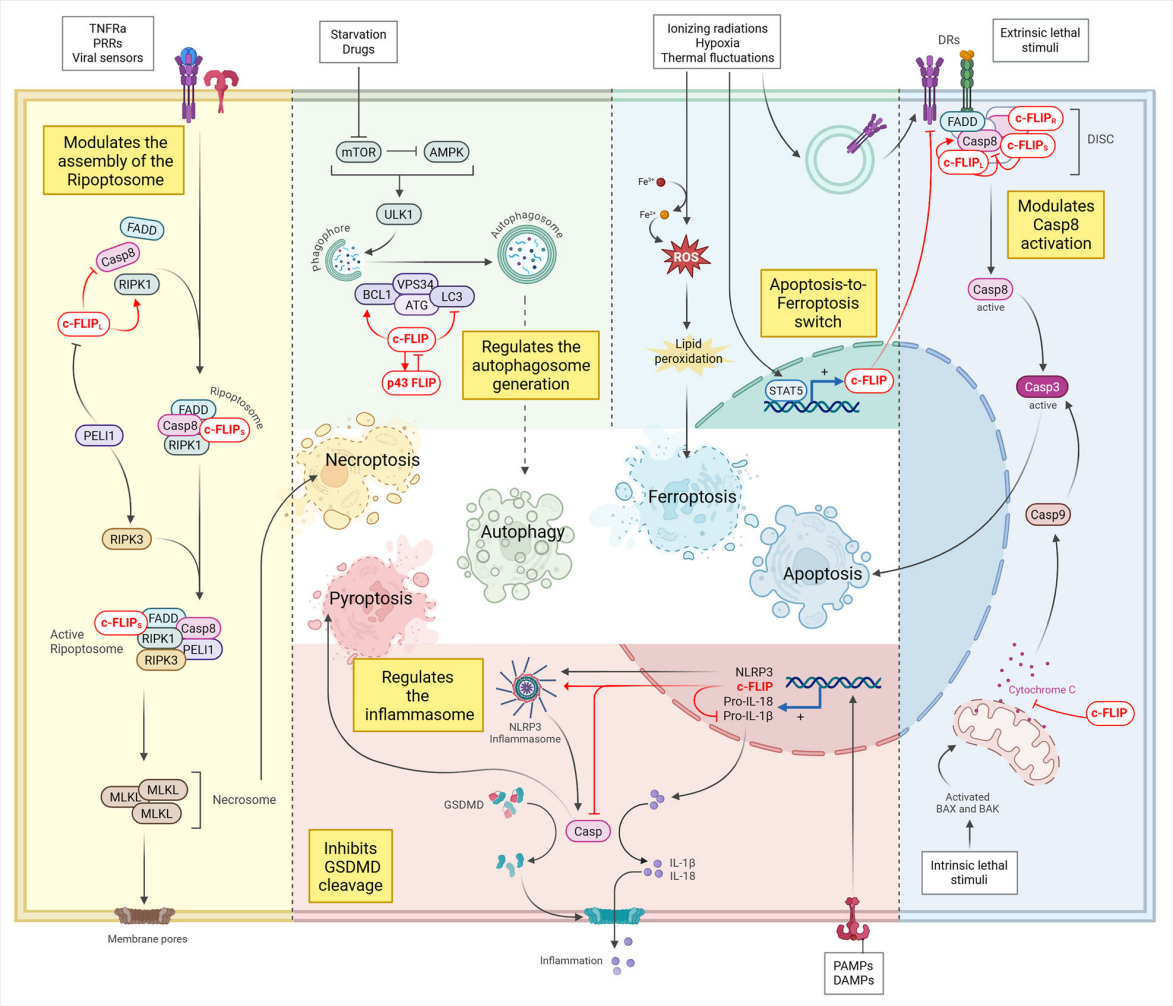
The role of c-FLIP in controlling cell death. c-FLIP is a crucial regulator of both apoptotic and non-apoptotic programmed cell death. *Necroptosis (yellow square)*. It is a caspase-independent pathway triggered by activation of members of the TNF receptor family (TNFRs), pattern recognition receptors (PRRs), and viral sensors. These receptor systems recruit proteins that activate receptor-interacting protein kinase 3 (RIPK3) and mixed lineage kinase domain-like protein (MLKL). Phosphorylated MLKL oligomerizes to form the necrosome. These MLKL oligomers then translocate to phosphatidylinositol phosphate (PIP)-rich domains in the plasma membrane, where they form large pores. The complex, defined as a ripoptosome, which includes receptor-interacting serine/threonine-protein kinase 1 (RIPK1), FADD, caspase-8, and c-FLIP, is considered a central inducer of necroptosis. High c-FLIP_L_ levels inhibit ripoptosome formation, while c-FLIP_S_ can promote necroptosis. Indeed, the protease-like domain of c-FLIP_L_ is essential for the activity of the caspase-8/c-FLIP_L_ heterodimer in blocking necroptosis. The caspase-8/c-FLIP_L_ heterodimer can cleave RIPK1, thereby inducing a blockade of necroptosis. *Autophagy (Green square)*. mTOR inhibition and AMPK activation induce formation of the ULK1 complex, followed by autophagosome assembly, which depends on Beclin-1 (BCL1), VPS34, LC3, and ATG proteins. c-FLIP can modulate autophagy and prevent cell death by interfering with ATG3-dependent LC3 interaction and processing, thus blocking autophagosome generation. Moreover, c-FLIPL can interact with Beclin-1, inhibiting its ubiquitination and proteasomal degradation, which is crucial for autophagosome nucleation. *Ferroptosis (Turquoise square).* Several stress signals, including ionizing radiation, hypoxia, and thermal fluctuations, can trigger Ferroptosis. Ferroptosis inducers can upregulate specific DRs that remain biochemically inactive after exposure to ferroptotic agents, potentially because these DRs associate with c-FLIP, which, in turn, can be upregulated by STAT5. Therefore, c-FLIP maintains the balance between ferroptotic and apoptotic pathways. *Pyroptosis (Red square).* The inflammasome complex, upon activation, leads to pyroptotic cell death, characterized by the production of IL-1β and IL-18. c-FLIP influences NOD-like receptor family pyrin domain-containing 3 (NLRP3) inflammasome assembly and activation by directly interacting with both NLRP3 and procaspases. In both monocytes and macrophages, stimulation of pattern recognition receptors (PRRs) from PAMPs and DAMPs induces the synthesis of pro-interleukin-1β (proIL-1β), proIL-18, and NLRP3, as well as c-FLIP. In this process, c-FLIP blocks caspase-8, thereby inhibiting activator proteolysis of pro-IL-1β, pro-IL-18, and of the pore-forming protein GSDMD, thereby inhibiting pyroptosis. *Apoptosis (Blue square).* *Extrinsic apoptosis*. It is triggered when cell-surface death receptors and dependence receptors (DRs) are activated by the presence or absence of extracellular stimuli. Upon activation, their intracellular domains serve as docking sites for the recruitment of the death-inducing signaling complex (DISC), which includes FADD, procaspase-8 (or procaspase-10), and various c-FLIP isoforms. Here, the stoichiometry of c-FLIP proteins precisely determines the life-or-death decision during death receptor signaling. In detail, the ratio between specific unbound c-FLIP isoforms and procaspase-8/10 governs the formation and enzymatic composition of the resulting heterodimers. The procaspase-8 (or 10)/c-FLIPL complex retains localized catalytic activity and preferentially promotes apoptosis by supporting DED-mediated procaspase-8 oligomerization. In contrast, the procaspase-8/c-FLIPS complex is catalytically inactive and acts as a dominant inhibitor of procaspase-8 activation. *Intrinsic apoptosis*. It is activated when perturbations of intracellular or extracellular microenvironment cause pro-apoptotic proteins (BAX/BAK) to oligomerize on the mitochondrial outer membrane, increasing its permeability and causing leakage of pro-apoptotic factors such as cytochrome C. c-FLIP has been shown to suppress the release of cytochrome c from mitochondria and might act as a negative regulator of the intrinsic apoptosis pathway.

c-FLIP integrates death- and survival-signaling pathways to determine cell fate. In addition to apoptosis and unregulated necrosis, cells undergo regulated non-apoptotic death programs including necroptosis, pyroptosis, and ferroptosis [82, 83]. Pathways that activate apoptosis can also engage regulated necrosis in innate immune cells (PANoptosis) [84], underscoring extensive cross-talk among death modalities with c-FLIP as a central node (Fig. 2).

#### Apoptosis

In the current nomenclature [83], apoptosis proceeds via either the extrinsic or the intrinsic pathway. Extrinsic apoptosis is initiated by TNFR superfamily death receptors (e.g., FasR/CD95, TRAIL-R1/R2), whose death domains recruit adaptors such as FADD and TRADD to assemble the DISC. Oligomerization and post-translational modifications (e.g., S‑palmitoylation of TRAILR1/FasR; O‑glycosylation of TRAIL receptors) facilitate receptor clustering into lipid rafts and formation of signaling protein oligomerization transduction structures (SPOTS), enabling caspase activation [85–87]. CD95 signaling requires internalization via “receptosomes,” whereas TRAIL-R1/R2 can signal at the plasma membrane [86, 87]. DISC composition is receptor-dependent but consistently contains FADD and procaspase‑8; ligand engagement recruits FADD via DED–DED interactions, which then brings procaspase‑8/‑10, TRADD, and c‑FLIP isoforms into the DISC. Proximity-driven dimerization triggers procaspase auto-cleavage; active caspase‑8 then cleaves caspase‑3 and Bid [83].

In TNFR1 signaling, TRADD rapidly nucleates Complex I with RIPK1, TRAF2, and cIAP1/2; FADD and procaspase‑8 join later after internalization [86]. c‑FLIP competes with procaspase‑8/‑10 for FADD binding within the DISC, modulating caspase‑8 dimerization/activation. Moreover, c‑FLIP and procaspase‑8 bind distinct FADD DED surfaces (α1/α4 vs α2/α5), with c‑FLIP showing higher affinity for α1/α4, thereby facilitating efficient DISC recruitment despite its lower cellular abundance [88]. NMR of c‑FLIP DED1 revealed canonical six‑helix folding and separable interfaces for FADD and calmodulin [89].

When death receptor signaling is triggered, following the assembly of RIPK1, FADD, and caspase-8 complex, caspase-8 and FADD form filamentous structures through the oligomerization of their tandem death effector domain (tDED), resulting in caspase activation and cell death. Indeed, the homotypic death domains of RIPK1 and FADD are not sufficient for their interaction, which requires the filamentation mediated by the DEDs of FADD (^FADD^DED) [90]. cFLIP can disrupt ^FADD^DED filaments, uncovering an additional antiapoptotic mechanism of cFLIP beyond its well-known function in disrupting caspase-8 filament. Molecular dynamics simulations revealed that ^FADD^DED filaments favor the incorporation of caspase-8 tDED monomers over ^FADD^DED monomers through electrostatic interactions, explaining the hierarchical assembly and stoichiometry of the FADD/caspase-8 complex [90].

Isoform-specific effects explain c‑FLIP’s paradoxical pro‑ and anti‑apoptotic roles. All isoforms harbor tandem DEDs that target the DISC. Short isoforms (c‑FLIP_S_, c‑FLIP_R_) fully block procaspase‑8 activation and apoptosis [11, 91–93]. p43‑c‑FLIP_L_ (lacking p12) likewise inhibits procaspase‑8 cleavage [92]. By contrast, c‑FLIP_L_ can promote initial procaspase‑8 processing via heterodimerization, yielding the p10 subunit and partial activation [11, 91–94]. Physiologic c‑FLIP_L_ levels enhance procaspase‑8 processing at the CD95‑DISC and promote apoptosis, whereas lower levels inhibit it [94]. Systems modeling showed that CD95 outcomes depend on the c‑FLIP_L_ vs c‑FLIP_S/R_ balance: moderate c‑FLIP_L_ (especially with strong receptor activation or abundant c‑FLIP_S/R_) augments caspase‑8 activation; high c‑FLIP_L_ or predominance of short isoforms suppresses the DISC [91]. Mouse genetics further supports these principles: in the wild‑derived MSM strain, a 21‑bp insertion in *Cflar* biases splicing toward c‑FLIP_L_ in hepatocytes, conferring resistance to FasR‑mediated liver failure by stabilizing caspase‑8 in the p43 state and blocking full p10/p20 maturation[95]. A reconstituted DISC system subsequently revealed that c‑FLIP isoforms are recruited cooperatively in a procaspase‑8–dependent manner; procaspase‑8/c‑FLIP_L_ heterodimers are pro‑apoptotic, whereas procaspase‑8/c‑FLIP_S_ heterodimers are inactive, with cell fate dictated by isoform ratios rather than simple competition [96].

Intrinsic apoptosis is triggered by intra/extracellular stresses (DNA damage, ER stress, replication or mitotic stress) that culminate in mitochondrial outer‑membrane permeabilization and cytochrome c release. c‑FLIP can negatively regulate the mitochondrial pathway in T cells, where c‑FLIP limits cytochrome c release; Bim deletion, but not caspase‑8 inhibition, rescues apoptosis in c‑FLIP‑deficient T cells, implicating c‑FLIP as a negative regulator of intrinsic apoptosis [97]. In cell lines, FADD elevation disrupts mitochondrial integrity and redox balance; combined c‑FLIP_L_ knockdown and FADD overexpression drives ROS/JNK1 activation and apoptosis, whereas c‑FLIP_L_ overexpression blunts intrinsic apoptosis induced by etoposide or HA14‑1 [98]. In an Alzheimer’s model, PS1‑induced apoptosis correlates with γ‑secretase–mediated c‑FLIP degradation [99]. The detailed cascades connecting c‑FLIP to intrinsic apoptosis remain incompletely defined.

#### Necroptosis

Necroptosis is a caspase‑independent, regulated necrosis initiated by TNFR family receptors, pattern‑recognition receptors, and viral sensors [82, 83, 100]. RHIM‑containing adaptors drive RIPK3 activation and MLKL phosphorylation/oligomerization; IP6 promotes MLKL oligomerization and pore formation in PIP‑rich membrane domains, leading to ionic imbalance and lytic death. RIG‑I and cGAS/STING can amplify necroptosis via type I IFN and TNF‑α feedback [101, 102]. Inhibitor of apoptosis proteins (IAPs) initially ubiquitylate RIPK1; CYLD deubiquitylates RIPK1 upon death signals, enabling RIPK3 recruitment and necrosome assembly [4, 82, 83, 100, 103–105]. c‑FLIP critically tunes necroptosis. The RIPK1–FADD–caspase‑8–c‑FLIP “ripoptosome” promotes RIPK1‑dependent death and was identified in 2011 [4, 103, 106]. Low RIPK1 or high c‑FLIP_L_ levels resist ripoptosome formation, whereas c‑FLIP_S_ can favor necroptosis in IAP‑deficient contexts [103, 106]. Pellino 1 (PELI1), an E3 ligase, stabilizes RIPK1–RIPK3 interactions and supports necrosome function via K63 ubiquitination of RIPK1, but its loss diminishes c‑FLIP_L_ and sensitizes cells to apoptosis [107]. The pseudo‑protease domain of c‑FLIP_L_ is essential for caspase‑8/c‑FLIP_L_ heterodimeric cleavage of RIPK1; Y362 is critical for procaspase‑8 activation and suppression of necroptosis [108].

#### Pyroptosis

Inflammasome‑dependent pyroptosis proceeds via a canonical caspase‑1 pathway or a non‑canonical caspase‑4/‑5/‑11 pathway [109, 110]. Inflammasome activation cleaves pro‑caspase‑1, which drives maturation of IL‑1β/IL‑18 and pyroptotic death [109–111]. c‑FLIP_L_ promotes the NOD-like receptor family pyrin domain-containing 3 (NLRP3) inflammasome assembly/activation by directly interacting with NLRP3 and procaspase‑1 and facilitating mitochondrial localization of NLRP3; c‑FLIP haploinsufficiency reduces IL‑1β production after adenosine triphosphate (ATP) or monosodium urate (MSU) crystal stimulation [112]. c‑FLIP also modulates AIM2 inflammasomes and associates with activated NLRP3 complexes via hnRNPK [112, 113]. In retinal neurons, reduced c‑FLIP limits the generation of gasdermin D N-terminal fragments (GSDMD-NT) and protects against pyroptosis [114]. Given pyroptosis’s roles in neurodegeneration and other diseases [115, 116], the therapeutic impact of targeting c‑FLIP requires deeper study.

#### Ferroptosis

Ferroptosis is an iron‑dependent form of cell death characterized by lipid peroxidation and mitochondrial changes [117, 118]. Ferroptosis inducers (e.g., sorafenib, erastin, artesunate) upregulate DR5 via ER stress pathways, initially suggesting synergy with TRAIL; however, these agents do not activate apoptotic signaling, and DR5 may remain inactive due to association with c‑FLIP [119]. In the gut, ferroptosis shapes the homeostasis of group 3 innate lymphoid cells (ILC3): GPX4 deletion reduces NKp46⁺ ILC3s, impairs IL‑22/IL‑17 A production, and worsens T cell‑mediated inflammation through the LCN2–p38–ATF4–xCT axis [120]. c‑FLIP is indispensable for IL‑7/IL‑15‑dependent NKp46⁺ ILCs (including NK cells and ILC3s): STAT5 activation precedes c‑FLIP upregulation, protecting developing cells from TNF‑induced apoptosis; conditional c‑FLIP loss ablates NKp46⁺ ILCs and drives early colitis with dysbiosis [121]. Together, these data suggest c‑FLIP safeguards NKp46⁺ ILCs by modulating the balance between ferroptosis and apoptosis.

#### Autophagy

The term “autophagy” originates from the Greek words “auto,” meaning “self,” and “phagein,” meaning “to eat”. Autophagy recycles intracellular constituents via lysosomal degradation and can either promote survival or contribute to cell death (autophagy‑dependent or autophagy‑mediated)[122, 123]. It buffers ROS and DNA stress in early tumorigenesis but can promote immune evasion and therapy resistance in advanced disease [124–126]. Mechanistically, mTOR inhibition and AMPK activation induce the formation of the Unc-51-like autophagy-activating kinase 1 (ULK1) complex, followed by Beclin1–VPS34 nucleation, ATG‑dependent elongation, LC3 lipidation, and SNARE‑driven autophagosome–lysosome fusion [127]. c‑FLIP restrains autophagy by disrupting ATG3–LC3 processing, thereby limiting autophagosome formation [5]. FLIP‑derived peptides that uncouple ATG3–FLIP–LC3 restore autophagy and cell death[128, 129]; primary c‑FLIP_L_‑deficient T cells show increased autophagy, apoptosis, and necroptosis [130]. Autophagy inducers can downregulate c‑FLIP_L_, thereby promoting apoptosis in cancer cells [131]. Conversely, c‑FLIP_L_ can stabilize Beclin‑1 by inhibiting its ubiquitination and proteasomal degradation [77]. Thus, c‑FLIP exerts bimodal, context‑dependent control over autophagy, motivating the development of agents that selectively perturb these interactions.

### Regulation of cell-signaling

**Fig. 3:**
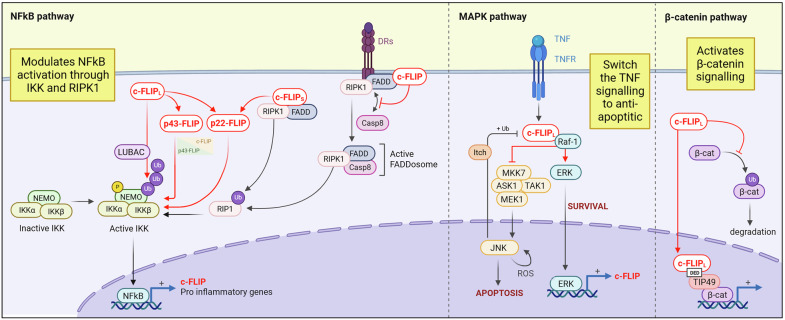
The role of c-FLIP in regulating cell signaling. c-FLIP plays a crucial role in regulating the balance between apoptotic and survival signals by controlling key pro-survival signaling pathways, including the NF-κB, MAPK, and Wnt pathways. In NF-κB signaling, the c-FLIP isoforms (c-FLIP_L_, p-43-FLIP, c-FLIP_S_, and p22-FLIP) can enhance the activation of IκB kinase (IKK). Specifically, these isoforms require the C-terminal domain of NEMO/IKKγ to facilitate IKK activation. However, none of these isoforms form a stable complex with IKKγ. The activation of IKK by c-FLIP_L_ also depends on the linear ubiquitin chain assembly complex (LUBAC), which generates a ubiquitinated substrate that can interact with IKKγ. In contrast, c-FLIP_S_ and the p22-FLIP cleavage product form a signaling complex with FADD and receptor-interacting serine/threonine-protein kinase 1 (RIPK1). This interaction promotes the ubiquitination of RIPK1, which subsequently activates IKK. TRAIL and FasRL receptors can trigger inflammatory responses through the cytosolic FADDosome, where caspase-8 acts as a scaffold to facilitate the FADD/RIPK1-mediated activation of NF-κB. c-FLIP may be recruited to the FADDosome, where it inhibits FasR- or TRAIL-triggered NF-κB signaling by disrupting the proper assembly of the FADDosome and interfering with the association of FADD and RIPK1 with caspase-8. NF-κB activity plays a crucial role in inducing the transcription of various anti-apoptotic genes, including c-FLIP. In turn, c-FLIP can sustain NF-κB signaling by generating proteolytic fragments, specifically p43-FLIP and p22-FLIP, from c-FLIP_L_. However, when c-FLIP_L_ is expressed at high levels, the production of these cleavage products decreases, which inhibits the activation of pro-survival NF-κB. Additionally, c-FLIP has been shown to regulate the activity of mitogen-activated protein kinases (MAPK), such as c-Jun N-terminal kinase (JNK) and extracellular signal-regulated kinase (ERK). After stimulation with TNFα, c-FLIP_L_ directly interacts with the JNK activator MAP kinase kinase (MKK)7, preventing its association with MAP/ERK kinase kinase 1 (MEK1), apoptosis-signal-regulating kinase 1 (ASK1), and TGFβ-activated kinase 1 (TAK1). This interaction ultimately inhibits JNK activation, which is responsible for driving the positive feedback mediated by ROS. TNFα-mediated activation of JNK promotes the degradation of the c-FLIP protein by stimulating the E3 ubiquitin ligase Itch, which specifically ubiquitinates c-FLIP, leading to its proteasomal degradation. The JNK-mediated degradation of c-FLIP_L_ antagonizes NF-κB signaling during TNFα responses. Therefore, the balance between NF-κB and JNK signaling determines whether TNFα induces cell survival or cell death. Additionally, c-FLIP_L_ has been shown to modulate MAPK/ERK activation by interacting with Raf-1, thereby enhancing its kinase activity. Conversely, ERK activation increases c-FLIP expression. c-FLIP_L_’s nuclear translocation also regulates the Wnt/β-catenin pathway. Specifically, the death effector domain (DED) of c-FLIP_L_ directly interacts with TIP49, a nuclear cofactor that influences β-catenin’s transcriptional activity. Furthermore, c-FLIP_L_ inhibits the ubiquitination of β-catenin, promoting its accumulation in the nucleus.

c‑FLIP coordinates death‑receptor signaling with broader survival pathways. Through interactions with TRAF1/2, RIPK1, and Raf‑1, c‑FLIP can activate NF‑κB, MAPK, and Wnt signaling (Fig. 3) [6], functioning as a molecular switch that tunes apoptosis versus survival across inflammation, tumorigenesis, and immune differentiation.

#### NF-κB signaling

Early studies established c‑FLIP as a modulator of CD95‑induced NF‑κB [132, 133]. Seminal work demonstrating c–FLIP–mediated recruitment of receptor-interacting protein (RIP) and subsequent NF-κB pathway activation has been extensively reviewed over the past two decades [7, 93]. In detail, c-FLIP N-terminal cleavage products p43-c-FLIP and p22-c-FLIP strongly induce NF-kB [69, 134]. p43‑c‑FLIP arises at the DISC after CD95 stimulation and can engage IKK directly or via TRAF1/2 and RIP [134, 135]; p22‑c‑FLIP forms during DR‑independent stimulation and may interact with IKK [69]. Systems modeling supports that non‑apoptotic versus apoptotic outcomes depend on procaspase‑8 and c‑FLIP fragment levels [136–138]. Experimentally, intermediate‑to‑high local c‑FLIP_L_ at the DISC permits p43‑FLIP generation and NF‑κB activation, whereas saturation inhibits procaspase‑8 recruitment and curtails p43‑FLIP [80]. Cell‑type differences exist: in keratinocytes, c‑FLIP can inhibit TRAIL‑induced NF‑κB [135, 136], while in TRAIL‑resistant cancers, c‑FLIP suppresses caspase‑8 yet supports NF‑κB/ERK survival signaling [139]. Recent data show no stable c‑FLIP–IKKγ complex: c‑FLIP_L_, c‑FLIP_S_, and p22-FLIP fragment require the C‑terminal NEMO/IKKγ domain (aa 272–419) and its ubiquitin‑binding capacity to facilitate IKK activation [140]. Notably, in the case of c-FLIP_L_, IKK activation also depends on the linear ubiquitin chain assembly complex (LUBAC), which likely generates a ubiquitinated substrate that can interact with IKKγ [140]. Although the DED1 domains of human c-FLIP and viral v-FLIP/K18 share only 32.6% sequence similarity, structural analyses show that both proteins bind to the same region of NEMO (amino acids 227–248) via their DED1 domains. Comparative structural modeling reveals that c-FLIP and v-FLIP/K18 each possess a binding pocket engaging NEMO residues phenylalanine 238 (F238) and aspartic acid 242 (D242). In v-FLIP/K18, this pocket is formed by residues histidine 83 (H83), leucine 75 (L75), valine 52 (V52), and proline 54 (P54), whereas in c-FLIP, L75 and P54 are substituted by methionine 74 (M74) and glycine 53 (G53), respectively [81]. Significantly, the replacement of P54 with G53 in c-FLIP expands the surface area of the putative NEMO interaction site, increasing the volume of the binding pocket. Moreover, both complexes maintain a conserved hydrogen bond between the side chain of NEMO residue D242 and H83 (v-FLIP/K18) or H82 (c-FLIP). In contrast, a second hydrogen bond present in the v-FLIP/K18/NEMO complex, formed between tyrosine 90 (Y90) and D242, is absent in the c-FLIP/NEMO complex. All these findings support the notion that both c-FLIP and v-FLIP harbor an NEMO binding site, with subtle differences that may affect their interaction strength [81]. In contrast, c-FLIP can also inhibit NF-κB and cytokine production driven by the FADDosome in FasR/TRAIL signaling by disrupting the assembly of FADD and RIPK1 on caspase-8 [141]. Overall, NF‑κB promotes anti‑apoptotic genes, including c‑FLIP; c‑FLIP sustains NF‑κB via c‑FLIP_S_ and/or p43/p22 fragments, while excess c‑FLIP_L_ diminishes their production and dampens NF‑κB.

#### MAPK signaling

Loss of NF‑κB‑mediated survival permits TNFα to reduce c‑FLIP_L_, leading to sustained JNK and ROS accumulation; ectopic c‑FLIP_L_ prevents this [78]. Mechanistically, c‑FLIP_L_ binds the JNK activator MAP kinase kinase (MKK)7 to block its association with MAP/ERK kinase kinase 1 (MEK1), apoptosis-signal-regulating kinase 1 (ASK1), and TGFβ-activated kinase 1 (TAK1), selectively suppressing the prolonged, ROS‑amplifying phase of JNK. Reciprocally, TNFα‑induced JNK activates the E3 ligase Itch, which ubiquitinates and degrades c‑FLIP_L_; mice lacking JNK1 or Itch resist TNFα‑induced liver failure and lack inducible c‑FLIP_L_ degradation [73, 142]. Thus, JNK‑driven c‑FLIP_L_ loss tilts responses toward death and antagonizes NF‑κB. c‑FLIP also modulates ERK1/2 and p38 by restraining caspase‑8 at the DISC [143]. NF‑κB‑dependent c‑FLIP_L_ induction is required for TNFα‑driven ERK activation, where c‑FLIP_L_ acts as a Raf‑1 activator independent of Ras [144]. In intestinal epithelium, ERK activity regulates c‑FLIP_S_ expression and function: MEK1 silencing or ERK inhibition reduces c‑FLIP_S_, whereas NF‑κB, JNK, or p38 inhibition does not, potentially contributing to epithelial apoptosis in ulcerative colitis and tumorigenesis [145].

#### Wnt signaling

Emerging findings have demonstrated that c‑FLIP_L_ extends its functional repertoire beyond NF‑κB and MAPK signaling by modulating the Wnt/β‑catenin pathway. In conjunction with FADD, c‑FLIP_L_ inhibits β‑catenin ubiquitination, thereby enhancing its nuclear accumulation [146]. Structural analyses revealed that the c‑terminal region of c‑FLIP_L_ contains a bipartite nuclear localization signal (NLS) and an adjacent nuclear export signal (NES), enabling nucleocytoplasmic shuttling [147]. In the nucleus, the DED domain binds TIP49 to potentiate β‑catenin–dependent transcription (e.g., ITF‑2), promoting proliferation [148]. Collectively, these studies underscore a dual role for c‑FLIP_L_: not only a DISC-associated anti-apoptotic factor but also an active nuclear effector that influences transcriptional programs.

#### Notch signaling

Recent research has revealed a complex interaction between c-FLIP and the Notch signaling pathway, an evolutionarily conserved process that plays a crucial role in maintaining tissue homeostasis. In vascular smooth muscle cells, Notch3 enhances c-FLIP expression via ERK/MAPK pathway, conferring FasL resistance independently of RBP‑Jκ [149]. Moreover, ubiquitin E3 ligases, such as Mind Bomb 1 (Mib1) and Deltex1 (DTX1), fine-tune this regulatory network by modulating c-FLIP stability and its interaction with caspase-8. This interaction ultimately determines whether cells survive or undergo apoptosis. These findings highlight the crucial role of c-FLIP in pathological conditions, including tumor progression and vascular disease.

#### Cellular response to endoplasmic reticulum stress

c-FLIP modulates apoptosis and helps maintain the integrity of the endoplasmic reticulum (ER) and mitochondria during ER stress. ER stress. ER stress provokes early c‑FLIP downregulation to facilitate caspase‑8 activation at DISC‑like platforms, sensitizing cells to apoptosis; sustained c‑FLIP expression is cytoprotective [150, 151]. Conversely, sustained expression or overexpression of c-FLIP during ER stress suppresses caspase-8-mediated apoptotic signaling, providing cytoprotection. Interestingly, cells lacking c-FLIP, such as c-FLIP-knockout mouse embryonic fibroblasts, exhibit disrupted ER architecture and paradoxically show resistance to ER stress-induced apoptosis [152–154]. This resistance is associated with impaired activation of key branches of the unfolded protein response (UPR), particularly the PERK and IRE1 pathways. c‑FLIP‑null mouse embryonic fibroblasts (MEFs) show disrupted ER architecture yet resist ER‑stress apoptosis due to aberrant AKT signaling that blunts PERK/IRE1 arms of the UPR [153, 154]. c‑FLIP localizes to mitochondria‑associated ER membranes (MAMs) to help maintain ER structure, inter‑organelle communication, Ca^2+^ homeostasis, and metabolic adaptation; Cflar^‑/‑^ MEFs display elevated lipid biosynthesis and lipid droplet accumulation during starvation [153, 154]. Thus, c‑FLIP links death‑receptor inputs to stress‑response programs and organelle integrity.

### c-FLIP and the immune system

**Fig. 4:**
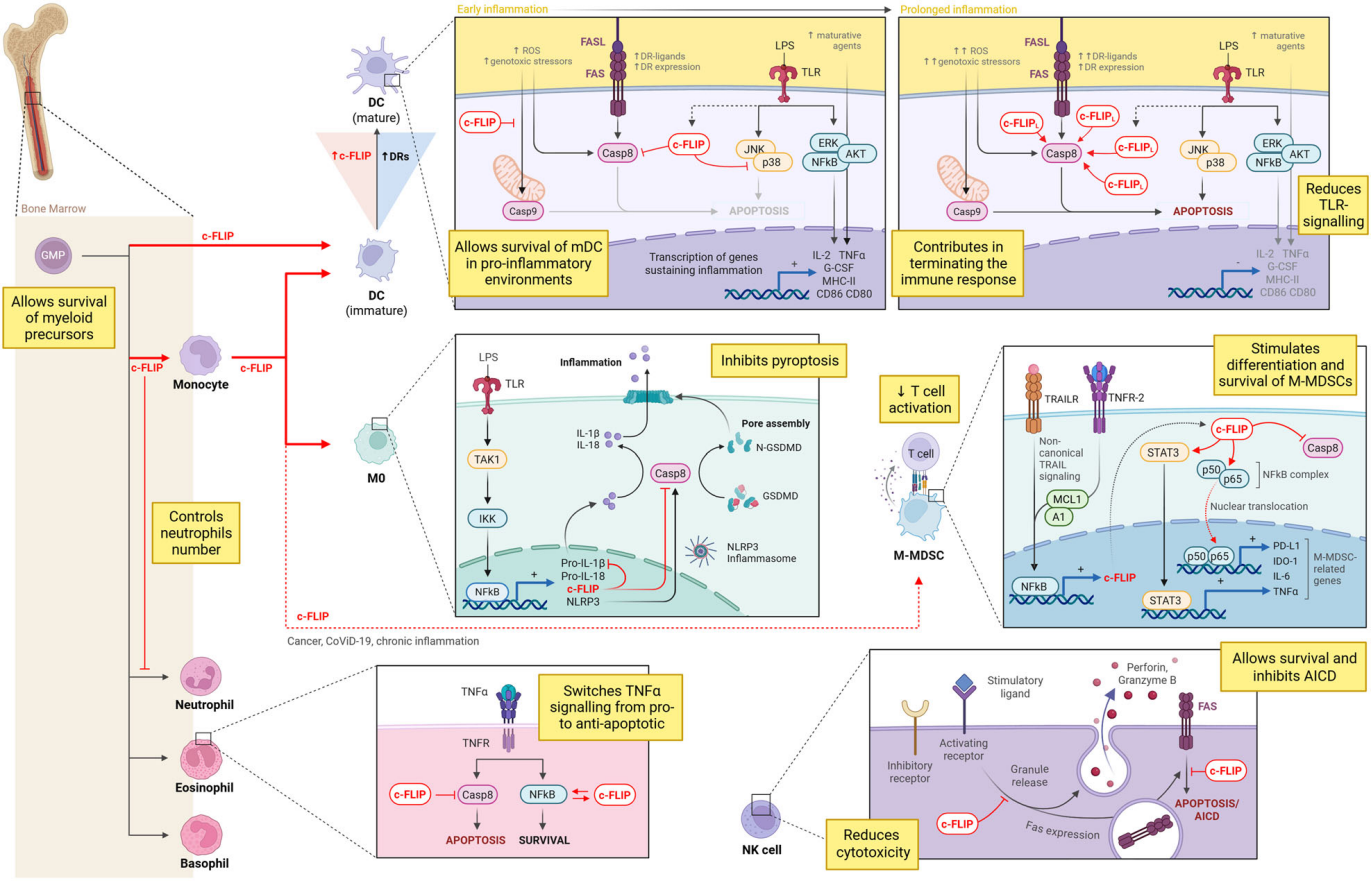
The role of c-FLIP in innate immunity. c-FLIP plays distinct roles across innate immune cell subsets. c-FLIP is necessary for the differentiation of DCs from bone marrow progenitors, independently of caspase inhibition. It is expressed at low levels in steady-state DCs and increases during maturation; this rise sustains the survival of mature DCs in inflammatory environments, characterized by high levels of death-receptor stimulators, ROS, and other genotoxic stressors, despite the parallel increase in death receptor expression by mature DCs. c-FLIP also acts in DCs as a negative modulator of activation signals transmitted by innate receptors, such as TLR2 or TLR4, antagonizing p38 MAPK activation and the subsequent release of pro-inflammatory cytokines TNF-α, G-CSF, and IL-2, and down-modulating MHC class II, and co-stimulatory molecules CD80 and CD86. Further increases in cFLIP_L_ levels during persistent inflammatory processes lead to caspase-8 cleavage and the induction of apoptosis as a feedback mechanism to terminate immune activation. c-FLIP is also indispensable for the survival of myeloid precursors and monocytes, and for their differentiation to macrophages. In both monocytes and macrophages, c-FLIP acts as a modulator of the intensity of the immune response, for example, by inhibiting pyroptosis. LPS stimulation of pattern recognition receptors (PRRs) induces the synthesis of pro-interleukin-1β (proIL-1β) and proIL-18, as well as of the NLRP3 inflammasome. In this process, active caspase-8 cleaves pro-IL-1β and pro-IL-18 into their active forms, and also cleaves GSDMD, resulting in the formation of membrane pores that facilitate the release of these pro-inflammatory cytokines, concurrent with cell death. In the presence of sufficiently high c-FLIP levels, caspase 8 action and, consequently, pyroptosis, are inhibited. c-FLIP expression following TLR-4 stimulation is rapidly induced in both monocytes and macrophages by TAK-1-mediated activation of downstream IκB kinase, thereby promoting the expression of NF-κB target genes. Notably, c-FLIP also partially inhibited pro-IL-1β expression. In several pathological conditions, c-FLIP has become a driver of the differentiation of immunosuppressive myeloid subsets that significantly inhibit T cell activation and closely resemble monocytic-myeloid-derived suppressor cells (M-MDSCs). The major drivers of M-MDSC differentiation and c-FLIP expression in myeloid cells are TNFR-2- and non-canonical TRAIL signaling, which lead to the activation of NF-κB. The extrinsic apoptotic pathway inhibitor MCL-1, or the MCL-1-related protein A1, is required for TNF-mediated c-FLIP expression. c-FLIP confers M-MDSC resistance to TRAIL- and TNF-mediated apoptosis by inhibiting caspase-8, thereby favoring their accumulation, and drives the establishment of immunosuppression-associated features, including the expression of PD-L1, IDO1, IL-6, and TNF-α, as well as inflammatory cytokines. This action is exerted through activation of STAT3 signaling and, by promotion of nuclear translocation of p65 and p50, of canonical NF-kB signaling. Although c-FLIP is not directly required for granulopoiesis, it maintains neutrophil homeostasis by sustaining myelopoiesis, which counterbalances abnormal granulocytic expansion. c-FLIP acts in eosinophils as a molecular switch, converting TNF-α signaling from pro-apoptotic to pro-survival, thereby mediating the inhibition of downstream caspase-8 activation and stimulation of NF-κB signaling. In NK cells, elevated c-FLIP levels diminish NK cell-mediated cytotoxicity triggered by stimulation of their activation receptors. Currently, c-FLIP plays a dominant role in inhibiting FasR-mediated apoptosis and activation-induced cell death (AICD), thereby influencing overall immune homeostasis.

**Fig. 5:**
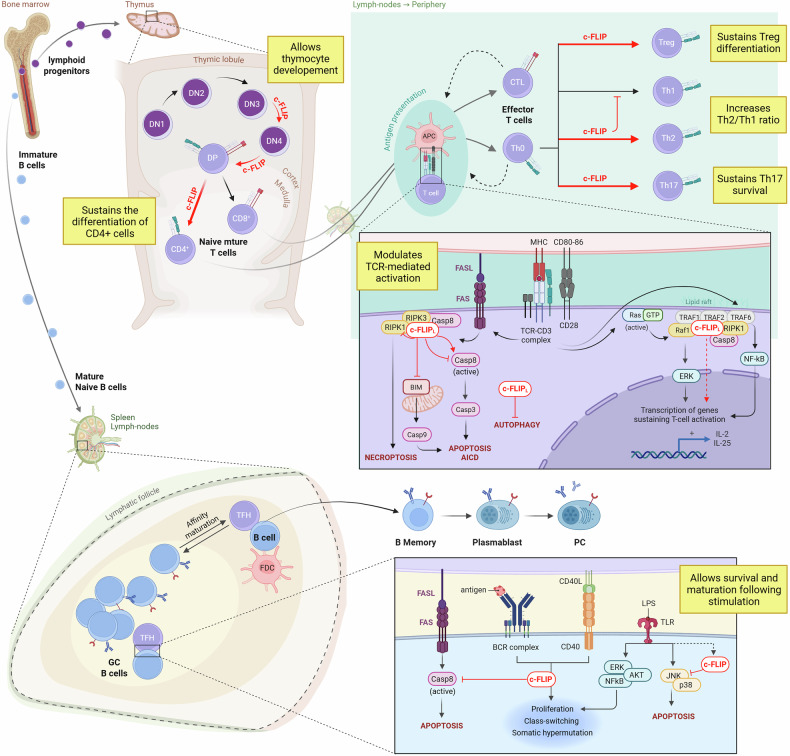
The role of c-FLIP in adaptive immunity. In adaptive immunity c-FLIP is essential for the differentiation of thymocytes, particularly following the double-negative (DN)-3 stage of development, and for the survival of mature, naïve T lymphocytes. In mature, resting T lymphocytes, it inhibits both the extrinsic and, through BIM, intrinsic apoptotic pathway, also counteracting activation-induced cell death (AICD); it reduces autophagy, and it binds the RIPK-1/RIPK3-necroptosis inducers, favouring their recruitment to the DISC, where caspase-8 can mediate inactivator cleavage of RIPK1, with consequent inhibition of necroptosis upon TCR stimulation. Moreover, c-FLIP provides a fundamental co-stimulatory signal to T cells: following TCR ligation, c-FLIP_L_ and caspase-8 rapidly co-localize to lipid rafts, where c-FLIP_L_ favours the recruitment of adaptor proteins TRAF1, TRAF2, TRAF6, RIPK1, and Raf1; RIPK1 is responsible for NF-κB activation, whereas Raf1 leads to activation of ERK; activation of these pathways allows expression of IL-2 and of the CD25 alpha subunit of the interleukin-2 receptor (IL-2R). Notably, c-FLIP_L_ itself also contributes to the termination of T lymphocyte-mediated immune responses since it can increase caspase-8 activity through its weak proteolytic action. c-FLIP can influence the polarization of effector T cells. It has been observed that c-FLIP_L_ drives the acquisition of the Th2 phenotype, confers a high ability to resist activation-induced cell death (AICD) in Th17 cells, and appears to be essential for Treg differentiation and survival. c-FLIP is not indispensable during the development of bone marrow precursor B cells, which, following maturation, migrate to secondary lymphoid organs (spleen and lymph nodes) where they meet the antigens supplied by the circulation. Follicular T helper cells (TFH) and follicular DCs (FDC) express co-stimulatory molecules (such as CD40L) which allow B cell proliferation, immunoglobulin class switching, and somatic hypermutation in response to foreign-antigen recognition, leading to maturation of plasmablasts, plasmacells (PC), and memory B cells in the germinal centre (GC). c-FLIP is essential for sustaining the GC response induced by both toll-like receptors (TLRs) and B cell receptors (BCRs), by balancing the equilibrium between survival signals provided by BCRs and CD40L, and apoptotic signals conveyed by FasR, leading to caspase-8 activation. When CD40 and BCR signaling are triggered simultaneously, they induce resistance to FASR-mediated apoptosis by the strong upregulation of c-FLIP. Following LPS stimulation, TLR signaling can mediate survival signals, conveyed through the NF-κB, ERK, and AKT pathways, and induce apoptosis via the JNK/p38 MAPK pathways. cFLIP is crucial for inhibiting p38 and JNK MAPKs in B cells, thereby enabling the survival of B cells upon LPS stimulation.

c-FLIP functions as a master regulator of immune cell fate, balancing activation and death across both innate and adaptive compartments. By modulating caspase-8 activity and integrating death receptor and NF-κB signaling, c-FLIP coordinates immune homeostasis while preventing excessive inflammation or autoimmunity. Aberrant expression, whether deficient or overactive, disrupts this balance, contributing to immune deficiency, chronic inflammation, or tumor-associated immunosuppression. In this section, we will examine the crucial role of c-FLIP in maintaining immune homeostasis, fostering tolerance, and regulating the functional activity of both innate (Fig. 4) and adaptive (Fig. 5) immune components.

### Innate immunity

#### Dendritic cells

DCs are professional antigen-presenting cells (APCs) that orchestrate the adaptive immune response by capturing and presenting antigens to T cells [155]. During maturation, immature DCs lose their phagocytic activity, increase their migration to lymph nodes, and enhance antigen presentation by upregulating HLA molecules and costimulatory proteins [159]. These changes expose them to apoptotic stimuli within inflammatory environments rich in death ligands and reactive oxygen species [156]. The anti-apoptotic protein c-FLIP_L_ is minimally expressed in resting DCs but is strongly induced during maturation by proinflammatory mediators, such as LPS, and by antioxidants, such as vitamin C [14]. This induction protects mature DCs from TRAIL- and FasR-mediated killing during T cell engagement, enabling sustained antigen presentation and robust activation of antigen (Ag)-specific T cells [15–18].

Beyond its role in apoptosis inhibition, c-FLIP also shapes DC immunogenicity. Viral FLIP overexpression in bone marrow-derived DCs promotes maturation, NF-κB activation, and upregulation of CD80, CD86, and CD40, enhancing proinflammatory cytokine release (TNF-α, IL-12) and IFN-γ production by T cells [157]. However, endogenous c-FLIP acts as a negative regulator of excessive activation: partial deletion of c-FLIP in murine CD11c⁺ DCs augments TNF-α, IL-2, and G-CSF secretion after TLR2/4 stimulation, alongside elevated MHC-II and costimulatory markers [158]. These DCs become hyperstimulatory toward T cells but are also more sensitive to LPS-induced death, underscoring c-FLIP’s dual role in balancing activation and survival. Mechanistically, this phenotype correlates with enhanced p38 MAPK signaling, suggesting that c-FLIP restrains proinflammatory responses by modulating MAPK signaling [158].

Functionally, c-FLIP deficiency in CD11c⁺ cells leads to an autoimmune-like phenotype resembling rheumatoid arthritis (RA), characterized by inflammatory joint destruction, autoreactive CD4⁺ T cells and autoantibody production [23]. The *CFLAR* locus has been identified as an RA susceptibility allele [159]. Mechanistically, c-FLIP deficiency impairs DC differentiation from hematopoietic progenitors and disrupts thymic Treg development, thereby reducing immune tolerance [23]. Conversely, c-FLIP overexpression dampens DC-driven γδ T cell activation, though the underlying mechanism remains undefined [160]. These findings collectively highlight c-FLIP as a critical molecular checkpoint that maintains DC viability while balancing immune activation and tolerance.

#### Monocytes and macrophages

Monocytes and macrophages are central components of innate immunity, responsible for phagocytosis, inflammatory signaling, and tissue homeostasis [161–164]. Both c-FLIP_L_ and c-FLIP_S_ isoforms are crucial for their survival and differentiation, protecting myeloid cells from death receptor-induced apoptosis under steady-state or inflammatory conditions [165–167]. During monocyte-to-macrophage maturation, c-FLIP_L_ and c-FLIP_S_ expression rises, ensuring successful differentiation and FasR resistance [165, 168]. Conditional deletion of c-FLIP in myeloid cells causes severe hematopoietic imbalance, with loss of macrophages, neutrophil infiltration, and myeloid hyperplasia due to defective clearance of apoptotic cells [169, 170]. This phenotype illustrates c-FLIP’s essential contribution to macrophage-mediated homeostasis [165, 168].

Functionally, c-FLIP fine-tunes inflammatory signaling and cell death pathways, particularly pyroptosis. In macrophages, LPS stimulation activates caspase-8 and NLRP3 inflammasome assembly, leading to IL-1β and IL-18 processing and GSDMD cleavage [19, 20]. High levels of c-FLIP_L_ inhibit full caspase-8 activation, thereby limiting pyroptosis and promoting survival [20]. Conversely, loss of c-FLIP_L_ sensitizes macrophages to LPS-induced death, while c-FLIP_S_ selectively dampens IL-1β release and NF-κB activation, highlighting isoform-specific control over inflammatory outputs [171]. Through these mechanisms, c-FLIP acts as a molecular rheostat linking pathogen recognition, cytokine release, and regulated cell death.

Pathologically, dysregulated c-FLIP expression contributes to chronic inflammation and tumor-associated immunosuppression. In cancer, TNF released in the tumor microenvironment (TME) induces NF-κB–dependent c-FLIP expression via TNFR2 signaling, preventing caspase-8 activation and extending myeloid-derived suppressor cells (MDSC) survival [172–174]. This mechanism underlies the accumulation of monocytic MDSCs (M-MDSCs), which suppress antitumor T cell responses [172, 175, 176]. GM-CSF–induced MCL-1 and A1 cooperate with TNF to sustain c-FLIP upregulation [175]. Consequently, M-MDSCs become resistant to apoptosis and maintain chronic inflammation.

In human monocytes, increased c-FLIP long and short isoforms drive immunoregulatory phenotypes by activating NF-κB and STAT3, promoting PD-L1, IDO1, IL-6, and TNF-α expression [53, 54]. These monocytes suppress T cell proliferation and favor Treg expansion in vitro and in vivo [54]. Elevated c-FLIP⁺PD-L1⁺CD14⁺ monocytes in PDAC and NSCLC patients correlate with poor outcomes [54, 177]. In COVID-19, excessive c-FLIP expression in monocytes activates a pathogenic STAT3 pathway, contributing to cytokine release syndrome and multiorgan inflammation [53]. Thus, while c-FLIP supports myeloid survival under physiological conditions, its persistent activation in pathological contexts drives immunosuppressive and inflammatory diseases.

#### Granulocytes

Granulocytes are a heterogeneous group of innate immune cells characterized by cytoplasmic secretory granules. This category includes neutrophils, eosinophils, basophils, and mast cells [178, 179]. Unlike in other myeloid cells, c-FLIP does not appear to be essential for neutrophil survival under steady-state conditions, as neutrophils have a short lifespan regulated mainly by intrinsic mitochondrial apoptosis pathways [169, 175]. While protein expression data indicate that c-FLIP is present in basophils, comprehensive functional studies specifically examining its role in these cells are currently lacking [180]. In eosinophils, c-FLIP shifts TNF-α signaling from pro-apoptotic to pro-survival by blocking caspase-8 activation and promoting NF-κB feedback [181]. In mast cells, c-FLIP upregulation following FcεRI or FcγR activation prevents FasR-mediated apoptosis, supporting their persistence in allergic inflammation [182]. Conversely, infection-induced downregulation of c-FLIP_S_ sensitizes mast cells to apoptosis, limiting chronic activation [183]. Natural Killer cells. Natural Killer cells (NKs) are innate lymphocytes that play a crucial role in defending the host against infections and cancer [184, 185]. In NK cells, c-FLIP_L_ prevents AICD and extends NK cell lifespan [186]. High c-FLIP expression in malignant NK cells, as seen in extranodal NK/T-cell lymphoma, confers FasR resistance, whereas c-FLIP knockdown restores sensitivity to apoptosis [186]. Beyond survival, c-FLIP overexpression can modulate NK cell cytotoxicity. Elevated c-FLIP levels can also reduce NK-mediated cytotoxicity, illustrating a trade-off between survival and effector function [187].

### Adaptive immunity

#### T lymphocytes

T cells are central to adaptive immunity, and c-FLIP is indispensable for their development, activation, and survival [188]. Acting as a checkpoint between death and survival signaling, c-FLIP regulates thymic maturation, TCR-induced activation, effector differentiation, and peripheral homeostasis. Both c-FLIP_S_ and c-FLIP_L_ isoforms contribute to T cell viability, as conditional deletion of c-FLIP in mature lymphocytes results in profound apoptosis and depletion of naïve T cells [189]. Beyond inhibiting death-receptor–mediated apoptosis, c-FLIP modulates intrinsic and autophagic pathways; its loss enhances BIM-dependent death, ROS generation, and autophagy [97, 106, 130]. Upon TCR stimulation, c-FLIP_L_ and caspase-8 translocate to lipid rafts, where limited cleavage of c-FLIP_L_ generates p43-FLIP. This fragment recruits which recruits RIPK1, TRAF2, and TRAF6, initiating NF-κB activation and supporting T-cell proliferation[190–192]. Thus, c-FLIP bridges apoptotic and pro-survival signaling, promoting both activation and longevity.

During thymic development, c-FLIP ensures the transition of thymocytes from double-negative (DN) to double-positive (DP) and finally to mature CD4⁺ and CD8⁺ single-positive (SP) subsets [193, 194]. Loss of c-FLIP arrests development at the DP stage, mirroring FADD- or Casp8-deficient phenotypes [195–197]. The severe depletion of SP thymocytes in these models reflects defective positive selection and survival. Differences observed between studies are primarily attributable to the timing of Cre-mediated deletion under the Lck promoter, active from early DN stages [194, 198, 199]. Overall, c-FLIP is essential for late thymic maturation rather than early lineage commitment.

Early studies revealed that death receptor engagement could costimulate T-cell activation in conjunction with TCR crosslinking [200, 201]. Although T cells lacking FasR or TNFR1 proliferate normally [200, 201], pharmacologic or genetic inhibition of caspase-8 causes profound activation defects [202–205]. This led to the identification of c-FLIP as a critical mediator downstream of death receptors, preserving T-cell viability during activation. Conditional knockout of c-FLIP in αβ T cells markedly reduces peripheral T-cell numbers and proliferation after TCR stimulation. In contrast, γδ T cells remain largely unaffected, underscoring lineage-specific dependency [193, 194, 199]. Conversely, c-FLIP_L_ overexpression enhances proliferation and IL-2Rα (CD25) expression following weak TCR stimulation [206]. Consistent with FADD- or caspase-8–deficient phenotypes [195, 196, 205, 207], c-FLIP-null T cells produce less IL-2 and exhibit defective IL-2 responsiveness despite expected CD25 upregulation [193, 194, 199]. While c-FLIP_L_ augments ERK and NF-κB activation downstream of TCR signaling [206, 208], these pathways can still be engaged in its absence, indicating that c-FLIP amplifies but is not essential for MAPK and NF-κB signaling. Isoform balance adds further complexity. Mice lack the human c-FLIP_S_ isoform and instead express c-FLIP_R_, which shares overlapping but distinct functions. c-FLIP_S_ competes with c-c-FLIP_L_ for caspase-8 binding, restraining NF-κB activation, and favoring apoptosis [194], while its complete loss also enhances death [9]. Conversely, c-FLIP_R_ overexpression drives hyperactivation and lupus-like autoimmunity, leading to Fas-resistant T cells [9]. Hence, physiological isoform ratios are essential for immune equilibrium.

The spatial regulation of c-FLIP–caspase-8 activity is crucial for balancing activation and death. Caspase-8 levels remain stable during activation, but its activity is confined to effector T cells [209]. Upon TCR stimulation, c-FLIP_L_ and caspase-8 relocate to lipid rafts, where caspase-8 cleaves c-FLIP to p43–FLIP, recruiting RIPK1, TRAF2, and TRAF6, and forming a platform that activates NF-κB [210]. The p43-FLIP fragment binds TRAF2 and RIPK1 with higher affinity than full-length c-FLIP [134, 135]. Upstream activators of NF-κB, including PKCθ, CARMA1, BCL-10, and MALT1, associate with this complex, and caspase-8 silencing disrupts MALT1/BCL-10 recruitment and NF-κB activation [210]. Thus, c-FLIP–caspase-8 complexes within membrane microdomains integrate apoptotic and pro-survival cues. c-FLIP also limits AICD, the FasR-dependent process that terminates T-cell responses and enforces tolerance [211]. Transgenic overexpression of c-FLIP suppresses AICD both in vitro and in vivo [189, 199], highlighting its role in sustaining effector expansion. However, excessive c-FLIP can paradoxically reduce long-term T-cell persistence. Although c-FLIP_L_-transgenic T cells resist FasR-mediated death, they exhibit increased basal caspase activity and accelerated turnover, leading to rapid loss of effector and memory cells after antigenic challenge [135, 206, 212]. Thus, c-FLIP serves as a rheostat, whose moderate expression supports activation and survival, whereas excessive levels precipitate premature apoptosis.

c-FLIP further shapes helper T-cell differentiation. Overexpression of c-FLIP_L_ favors Th2 polarization with increased IL-4 and reduced IFN-γ production [213, 214], whereas silencing enhances T-bet and IFN-γ, promoting Th1 commitment [215]. IL-4/STAT6 signaling induces c-FLIP_S_, establishing a feedback loop that reinforces Th2 identity [233]. Th17 cells also exhibit elevated c-FLIP, conferring resistance to Fas-mediated apoptosis and persistence in chronic inflammation [216].

Tregs critically depend on c-FLIP for survival. Treg-specific deletion of *CFLAR* triggers a fatal form of autoimmunity reminiscent of FoxP3 deficiency [22, 217]. Although Tregs express lower levels of c-FLIP_S_ than conventional T cells [9], residual expression prevents spontaneous apoptosis and maintains suppressive function beyond Fas-mediated regulation [22]. In ovarian cancer, either c-FLIP_L_ or c-FLIP_S_ overexpression protects tumor-infiltrating Tregs from FasL-mediated death, enhancing immune suppression [218]. Furthermore, p53 loss upregulates c-FLIP_L_, strengthening death resistance in both malignant and Treg subsets [219, 220].

Finally, the relevance of c-FLIP extends to chimeric antigen receptor (CAR)–T cells, which revolutionized cancer immunotherapy [221]. A significant limitation of CAR-T efficacy is AICD triggered in the TME [222, 223]. Because c-FLIP antagonizes AICD, enhancing its signaling may improve CAR-T persistence. Genetic or pharmacologic modulation of c-FLIP pathways is being explored to prevent CAR-T exhaustion [224]. However, overexpression of p43-FLIP within CD28–HER2 CAR constructs partially inhibits FasR-mediated death but impairs effector function and antitumor activity in vivo [225]. These findings emphasize that precise tuning rather than a global enhancement of c-FLIP activity is necessary to optimize CAR-T cell performance.

B lymphocytes. B lymphocytes are key players in the adaptive immune response, whose primary role is to lead humoral immunity by producing antibodies against pathogens, thereby facilitating their rapid elimination [226]. During the differentiation phases, which occur first in the bone marrow and then in the spleen, immature B cells receive survival signals from the pre-BCR and BCR [227]. It has been shown that conditional deletion of c-FLIP in B cells from the pro-B stage does not affect the development of bone marrow precursor B cells [228, 229], consistent with previous findings that FasR signaling is dispensable for the development of immature B cells [230]. Then, mature B cells migrate to secondary lymphoid organs (spleen and lymph nodes), where they meet the antigens constantly supplied by the lymphatic circulation. The fate of mature B cells depends on the balance between survival signals, provided by BCRs and CD40L, and apoptotic signals, conveyed by FasR and leading to caspase-8 activation [227, 231].

When CD40 and BCR signaling are triggered simultaneously, they confer resistance to FasR-induced apoptosis by strongly upregulating c-FLIP_L_, a response that persists for approximately 24 h [230, 232]. A deep analysis performed on mice with conditional deletion of c-FLIP in the B lineage (c-FLIP^f/f^; CD19Cre mice) surprisingly revealed that B cells lacking c-FLIP have not only hypersensitivity to FasR-induced apoptosis and impaired proliferative responses induced by the BCR, but also fail TLR-induced proliferation [228], leading to a reduced amount of circulating mature B cells [230, 232]. Indeed, c-FLIP-defective B cells exhibited aberrant phenotypic features, producing significantly reduced levels of both IgM and IgG2a antibodies, and were defective in class switching in response to immunization with either T cell-independent or T cell-dependent antigens [230, 232]. Collectively, these data indicate that c-FLIP is essential for sustaining the GC response induced by both TLRs and BCRs; however, further studies are needed to elucidate the molecular pathways underlying these regulatory feedback loops.

### c-FLIP signaling in human diseases

**Fig. 6:**
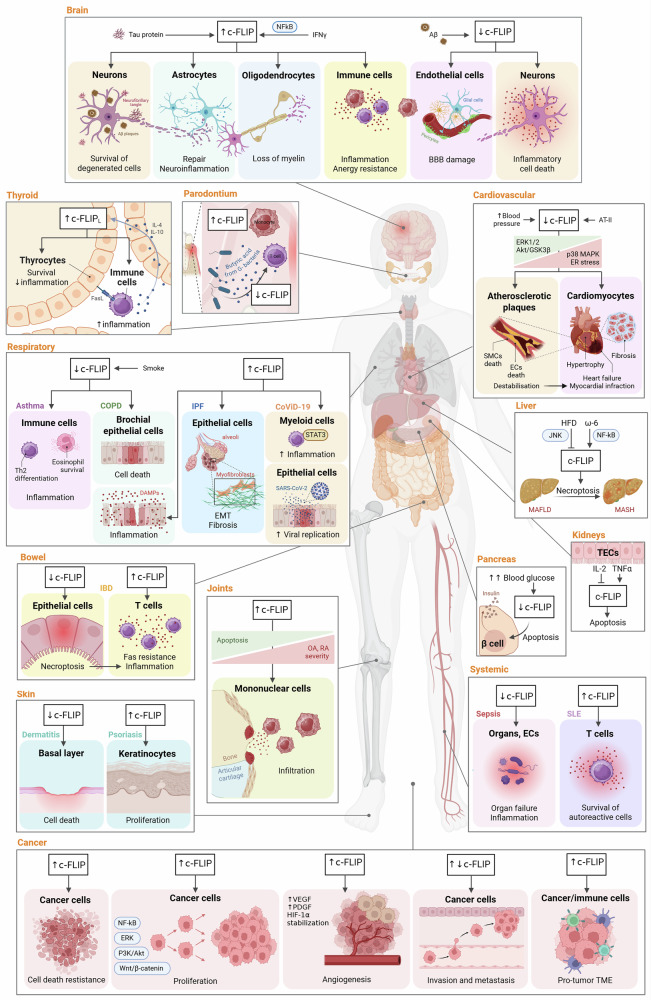
The role of c-FLIP in pathologies. Multiple findings have elucidated the role of FLIP in various diseases. In the CNS, c-FLIP reduction in neurons is strongly correlated with inflammatory neuronal cell death in several pathologies. In other diseases, such as Alzheimer’s disease, pathogenic tau proteins (which form neurofibrillary tangles) induce c-FLIP overexpression in dysfunctional neurons, thereby favoring their survival and disease progression. c-FLIP is essential for protecting oligodendrocytes from inflammatory cell death that occurs in multiple sclerosis. In this context, IFN-γ triggers a protective mechanism that induces c-FLIP in these cells via NF-κB activation. Furthermore, an increase of c-FLIP in reactive astrocytes and microglial cells near injury sites has been reported, suggesting a role in brain repair, glial scar formation, and neuroinflammation. c-FLIP also regulates the survival of brain vascular endothelial cells and pericytes surrounding cerebral capillaries, which is fundamental to the integrity of the blood-brain barrier (BBB). Several pathologic stimuli (such as amyloid β) reduce c-FLIP in these cells. In multiple sclerosis, high c-FLIP in T cells and innate immune cells also contributes to neuroinflammation. In Periodontitis, butyric acid produced by Gram-negative bacteria downregulates c-FLIP expression in T cells, thereby increasing their susceptibility to cell death. Conversely, c-FLIP is upregulated in monocytes, fueling inflammation. In the context of inflammatory pathologies of the thyroid (such as Hashimoto’s thyroiditis and Graves’ disease), high c-FLIP in infiltrating inflammatory cells contributes to the establishment of chronic inflammation; whereas c-FLIP overexpression in thyrocytes (which can be promoted by the Th2 cytokines IL-4 and IL-10), protects them from inflammatory damage both directly and indirectly, driving FasRL-apoptosis in immune cells. Myocardial stressors, such as pressure overload or neurohormonal stimulation (e.g., angiotensin (AT) II), downregulate c-FLIP expression in cardiomyocytes. Decreased c-FLIP triggers pro-apoptotic and pro-fibrotic pathways, including ER stress and p38 MAPK signaling, and suppresses survival signaling pathways such as Akt/GSK3β and ERK1/2. This leads to cardiac hypertrophy and fibrosis, which can lead to heart failure and myocardial infarction. Loss of c-FLIP also favors ischemic cardiac disease by destabilising the atherosclerotic plaques. Here, c-FLIP expression is often reduced or uneven, especially in smooth muscle cells (SMCs) and endothelial cells (ECs), which display increased apoptosis. Cigarette smoke downregulates c-FLIP in bronchial epithelial cells, leading to cell death and inflammation. In established Chronic obstructive pulmonary disease (COPD), c-FLIP increases and skews the balance toward necroptosis, resulting in the release of damage-associated molecular patterns (DAMPs) and exacerbation of airway inflammation. Decreased c-FLIP expression has been reported in PBMCs of severe asthmatic patients; currently, c-FLIP promotes the differentiation of Th2 cells and sustains the survival of eosinophils, favouring allergic reactions. In patients with idiopathic pulmonary fibrosis (IPF), c-FLIP is distinctly upregulated in alveolar epithelial cells and myofibroblasts within areas of fibrosis, likely contributing to the survival of epithelial cells undergoing epithelial–mesenchymal transition (EMT) and supporting the progression of fibrosis. c-FLIP is markedly upregulated during SARS-CoV-2 infection in both infected lung epithelium, allowing enhanced viral replication, and circulating myeloid populations, which activate T3-dependent hyperinflammatory state and cytokine release syndrome (CRS). In pancreatic beta cells, the expression of c-FLIP is impaired by high glucose concentration in diabetes patients. Since high glucose also upregulates FasR, the result is the death of beta cells. A high-fat diet (HFD) leads to JNK-mediated degradation of c-FLIP in hepatocytes, resulting in increased susceptibility of hepatocytes to apoptosis and necroptosis, which in turn fuels inflammation and progression toward MASH. On the contrary, a diet rich in ω-6 fatty acids upregulates FLIP through NF-κB signaling. c-FLIP is essential for maintaining renal tubular epithelial cell (TECs) homeostasis under physiological conditions and protects them from apoptosis in several acute and chronic kidney diseases. In pathological conditions, the cytokine milieu could promote (e.g., TNF-α) or repress (e.g., IL-2) c-FLIP expression, determining the fate of TECs. c-FLIP influences the course of inflammatory bowel diseases (IBD), which is often downregulated in intestinal epithelial cells (IECs), favouring necroptosis and, consequently, inflammation. On the contrary, high c-FLIP in infiltrating T cells has been detected, rendering them resistant to FasR-mediated apoptosis and fueling inflammation. c-FLIP is not expressed in normal synovium, whereas it is present in pathologies such as osteoarthritis (OA) and rheumatoid arthritis (RA), where the compromised apoptosis contributes to increased mononuclear infiltration in cartilage and bone, as well as in the synovium. c-FLIP was significantly higher in keratinocytes from patients with psoriasis, whereas it was decreased in the basal layer of the human epidermis in idiopathic dermatitis, disrupting epidermal cytoarchitecture. Reduction in the expression of c-FLIP has been observed in multiple organs in sepsis patients, exacerbating endothelial cell (ECs) dysfunction and sepsis progression. High c-FLIP is fundamental for sustaining prolonged survival of anergic autoreactive T cells in systemic lupus erythematosus (SLE), favouring both the establishment and progression of the disease. In tumors, c-FLIP up-regulation inhibits FasR-mediated apoptosis, promoting the resistance to targeted therapies and immune evasion processes. Beyond its role as anti-apoptotic molecule in cancer cell, c-FLIP strongly enhance NF-kB, PI3K/Akt, ERK, and Wnt/β-catenin pathways to support cell survival, proliferation, and immunosuppression within the tumor microenvironment (TME). Furthermore, c-FLIP trigger angiogenic programs by up-regulating vascular endothelial growth factor (VEGF), platelet-derived growth Factor (PDGF), and stabilizing the alpha subunit of transcription factor hypoxia-inducible factor-1 (HIF-1α) under hypoxia, thus increasing VEGF. The role of c-FLIP in invasion and metastasis is context-dependent. Some tumor tissues show a reduced c-FLIP expression in comparison to normal tissues, correlating with adverse outcomes and metastasis; in other contexts, c-FLIP isoforms are enriched in cancer cells and are significantly associated with metastasis.

c-FLIP is a master regulator of death-receptor (DR) signaling that integrates apoptotic and pro-survival cues to control cell fate. Aberrant expression or activity of c-FLIP perturbs tissue homeostasis and is implicated across oncology, autoimmunity, and chronic inflammatory disorders (Fig. 6). In tumors, constitutive c-FLIP inhibits FasR-mediated apoptosis, promotes resistance to genotoxic and targeted therapies, and facilitates immune evasion. Beyond its role in apoptosis blockade, c-FLIP engages NF-κB, PI3K/Akt, and ERK pathways to support proliferation, angiogenesis, and immunosuppression within the TME. In autoimmunity, dysregulated c-FLIP impairs the deletion of autoreactive lymphocytes and disrupts tolerance mechanisms. Accordingly, c-FLIP is emerging as both a biomarker and a therapeutic target; pharmacologic or genetic strategies that modulate c-FLIP abundance or function may restore apoptosis in cancer, recalibrate inflammatory circuits, and correct pathogenic cell-survival programs.

### Cancers

Global cancer incidence is projected to exceed 35 million new cases annually by 2050, a 77% increase versus ~20 million in 2022, driven by demographic aging/expansion and modifiable risks (tobacco, alcohol, obesity, environmental carcinogens)[233, 234]. Malignant transformation reflects the acquisition of hallmark capabilities, such as sustained proliferation, resistance to growth suppressors, evasion of apoptosis, replicative immortality, angiogenesis, invasion, and metastasis, underpinned by genetic and epigenetic rewiring [235–237]. c-FLIP intersects multiple hallmarks central to tumor initiation, progression, and therapeutic resistance.

#### Resisting cell death

As apoptosis evasion is foundational to tumor persistence under genotoxic stress, c-FLIP is frequently overexpressed across human malignancies. Elevated c-FLIP has been documented in colorectal colorectal [238–240], pancreatic [241, 242], gastric [243], hepatocellular [244], head and neck squamous cell carcinoma [245, 246], non-small cell lung cancer [247, 248], prostate [249, 250], ovarian [251, 252], and bladder carcinoma [253–255], melanoma [256–258], osteosarcoma [259], and glioblastoma [260, 261], as well as chronic lymphocytic leukemia [262, 263], Hodgkin’s lymphoma [264], and multiple myeloma [265, 266]. In most settings, the c-FLIP_L_ isoform predominates and correlates with poor outcomes and resistance to 5-fluorouracil, cisplatin, sorafenib, and gemcitabine [248, 257, 267–270]; c-FLIP_S_ isoform upregulation also occurs in subsets such as lung carcinoma [271] (Table 1). Functional genomics reinforces the context-specific role of c-FLIP: drug-induced senescent tumor cells upregulate c-FLIP and remain apoptosis-resistant despite DR5 induction, underscoring c-FLIP’s nonredundant protection at senescence [272, 273].

**Table 1:** Summary of c-FLIP involvement in cancer.

Cancer Type	Cancer name	c-FLIP expression	Hallmarks	References
Solid tumor	Lung Cancer	Up	Apoptotic resistance	[247, 248, 432]
	Colorectal Cancer	Up	Apoptotic resistance	[238–240]
	Melanoma	Up	Apoptotic resistance	[256–258]
	Breast Cancer	Up	Apoptotic resistance	[433]
	Prostate carcinoma	Up	Apoptotic resistance	[249, 250]
	Pancreatic carcinoma	Up	Apoptotic resistance	[241, 242]
	Glioblastoma	Up	Sensitization to apoptosis	[260, 261]
	Ovarian carcinoma	Up	Motility and invasion	[251, 252]
	Gastric carcinoma	Up	Apoptotic resistance, progression	[243]
	Hepatocellular carcinoma	Up	Apoptotic resistance	[244]
	Bladder carcinoma	Up	Apoptotic resistance	[253–255]
	Head and neck squamous cell carcinoma	Up	Apoptotic resistance	[245, 246]
	Renal cell carcinoma	Up	Apoptotic resistance	[393, 434]
	Urothelial carcinomas	Up	Apoptotic resistance	[253, 255]
	Cervical intraepithelialneoplasia/cervical carcinoma	Up	Apoptotic resistance	[303, 435]
	Thyroid cancer	Up	Apoptotic resistance	[436]
	Esophageal cancer	Up	Apoptotic resistance	[437]
	Endometrial carcinoma	Up	Apoptotic resistance	[438, 439]
	Thymic epithelial tumors	Up	Autophagy resistance	[440]
	Germ cell tumors	Up	Unspecified	[441]
	Meningiomas	Up	Apoptotic resistance	[442, 443]
	Retinoblastoma	Up	Apoptotic resistance	[444]
	Cholangiocarcinoma	Up	Apoptotic resistance	[445]
	Breast Cancer	Down	Loss of apoptosis, invasion	[283]
	Soft tissue sarcoma	Down	Regulation of immune response	[284]
Hematological malignancy	T-cell acute lymphoblastic leukaemia	Up	Unspecified	[446]
	Chronic myeloid leukemia	Up	Apoptotic resistance	[447]
	Diffuse large B-cell lymphoma	Up	Apoptotic resistance	[448]
	Follicular lymphoma	Up	Apoptotic resistance	[449]
	Osteosarcoma	Up	Apoptotic resistance	[259]
	Burkitt’s lymphoma	Up	Apoptotic resistance	
	Hogkin’s lymphoma	Up	Apoptotic resistance	[264]
	Acute myeloid leukaemia	Up	Apoptotic resistance	[450]
	Chronic lymphoid leukemia	Up	Apoptotic resistance	[262, 263]
	Multiple myeloma	Up	Apoptotic resistance	[265, 266]

#### Sustaining proliferative signaling

c-FLIP_L_ augments cell-cycle entry and growth via NF-κB, PI3K/Akt, ERK, and Wnt/β-catenin pathways, ultimately increasing cyclin and proto-oncogene levels [274, 275]. In lung and prostate cancer, c-FLIP_L_ stabilizes β-catenin by limiting its ubiquitination and proteasomal turnover, thereby elevating β-catenin–dependent targets such as cyclin D1 and promoting G1/S transition [148, 276]. c-FLIP_L_ can also cooperate with FADD to potentiate canonical Wnt signaling and sustain cyclin D1 [146]. Pharmacologic disruption of eIF4E–eIF4G reduces cyclin D and downregulates c-FLIP, linking translational control to c-FLIP abundance [277]. In the brain TME, an astrocyte–glioblastoma (GBM) axis mediated by ANXA1 (astrocytes) and FPR1 (GBM) suppresses immunogenic necroptosis by downregulating *CFLAR*, facilitating immune escape and tumor progression [278]. Notably, c-myc restrains c-FLIP transcription [56], forming a feedback that can re-sensitize cells to death-ligand therapies (e.g., TRAIL) when c-myc is high.

#### Inducing cancer angiogenesis

c-FLIP promotes angiogenic programs by upregulating vascular endothelial growth factor (VEGF) and auxiliary mediators [279]. c-FLIP_L_ and its cleavage product p43-c-FLIP activate NF-κB via TRAF2 and RIPK1 to enhance transcription of VEGF, IL-8, PDGF, and MMPs, supporting endothelial proliferation and neovascularization [141, 274, 279]. c-FLIP also engages ERK signaling by interacting with Raf-1 and upstream kinases [280], thereby promoting endothelial survival and cytoskeletal remodeling (sprouting) and stabilizing the alpha subunit of transcription factor hypoxia-inducible factor-1 (HIF-1α) under hypoxia, thereby further increasing VEGF [281, 282]. Although data in tumor endothelial cells/pericytes remain limited, high endothelial c-FLIP may modulate immune–vascular crosstalk, including protection of Tregs from FasR-induced apoptosis within the TME [218].

#### Activating invasion and metastasis

c-FLIP’s role in metastasis is context-dependent. In normal mammary epithelia, extracellular matrix (ECM) detachment induces c-FLIP to prevent anoikis; in breast cancer, oncogenic PI3K/Akt suppresses this compensatory c-FLIP induction during detachment, enabling anchorage-independent growth and metastatic potential [283]. Soft-tissue sarcoma exhibits reduced c-FLIP expression relative to matched normal tissues; this downregulation correlates with adverse outcomes yet functionally confers resistance to anoikis by limiting caspase-8 activation [283, 284]. Pharmacologic c-FLIP downregulation restores anoikis and curtails metastasis, nominating c-FLIP as a therapeutic lever to block dissemination [285]. Metastasis involves sequential detachment, invasion, intravasation, survival in circulation, extravasation, and colonization at distant sites [286, 287]. Cancer stem cells (CSCs) orchestrate these steps by driving EMT, migratory programs, and anoikis resistance while remodeling the TME to form supportive niches [288]. In GBM, c-FLIP isoforms are enriched in glioblastoma stem-like cells (GSCs) versus bulk tumor; c-FLIP silencing sensitizes GSCs to temozolomide-induced apoptosis [289]. Similarly, breast cancer stem cells (BCSCs) show elevated c-FLIP; knockdown compromises self-renewal and enhances apoptosis, highlighting translational opportunities to deplete relapse-seeding populations [290].

#### Altering the TME immune landscape by promoting inflammation and avoiding immune reaction

The TME comprises neoplastic, stromal, vascular, and diverse immune cells. Innate (macrophages, neutrophils, NK cells, DCs, MDSCs) and adaptive (T cells, including Tregs) populations can either constrain or support tumor growth through cytokine/chemokine networks [291, 292]. c-FLIP shapes this ecosystem at multiple levels. Tumors overexpressing c-FLIP resist CTL- and NK-mediated cytotoxicity via FasL/TRAIL pathways, allowing immune escape; enforced c-FLIP supports tumor establishment in immune-competent hosts by blocking FasR-dependent killing [24]. Infiltrating suppressor populations also depend on c-FLIP: MDSCs require c-FLIP to withstand DR-induced apoptosis and maintain suppressive function [175]; Tregs rely on c-FLIP for homeostasis and fitness [9]. Environmental and metabolic cues modulate c-FLIP expression. Oxidative stress derivatives such as peroxynitrite can upregulate c-FLIP, enhancing survival under redox duress [293]. In castration-resistant prostate cancer, elevated TNF-α associates with poor prognosis and co-expression of c-FLIP_L_ and c-FLIP_S_, which synergistically inhibit caspase-8 and confer TNF-α resistance [294]. In pancreatic ductal adenocarcinoma, high serum IL-6 correlates with increased c-FLIP⁺PD-L1⁺ immunosuppressive monocytes and reduced survival, suggesting an IL-6–c-FLIP–PD-L1 axis of immune escape [54]. c-FLIP also interfaces with type I interferon (IFN) pathways, which exert context-dependent antitumor or protumor effects [295]. c-FLIP_L_ suppresses type I IFN by binding IRF3 and preventing DNA binding and co-activator recruitment, thereby disrupting IFNβ enhanceosome assembly [296]. The same isoform also limits IRF7 activation downstream of TLR9 by sequestering IKKα and preventing IRF7 phosphorylation and dimerization [297]. In contrast, c-FLIP_s_ can promote type I IFN production by activating caspase-8 [298]. Thus, c-FLIP coordinates apoptotic and inflammatory arms of type I IFN signaling to influence antitumor immunity. Finally, metabolic states in the TME influence c-FLIP and cell-death sensitivity [299]. Caloric restriction (CR) dampens growth pathways (insulin/IGF-1, mTOR) and reduces c-FLIP, sensitizing malignant cells to apoptosis. CR combined with lysine-specific demethylase 1 (LSD1) inhibition downregulates c-FLIP and augments apoptosis in acute myeloid leukemia and breast cancer models, improving tumor control [300]. Conversely, glycolytic rewiring can shield tumors from CTL killing. Genome-wide CRISPR screens identified Glut1 (glucose transporter 1) and Gpi1 (glucose-6-phosphate isomerase 1) as mediators of resistance to CTL cytotoxicity; their loss reprograms metabolism toward oxidative phosphorylation, leading to increased ROS, which potentiates TNFα-mediated death by downregulating c-FLIP and activating caspase-8 [301]. These data expose therapeutic opportunities that couple metabolic intervention to death-receptor sensitization.

Based on the critical role of c-FLIP in tumorigenesis and development, accurate detection of its expression is of great significance for both basic research and clinical applications. Protein-level detection relies on immunohistochemistry (IHC) and western blotting (WB), which afford spatial and semi-quantitative information [302]. Transcriptional assessment by RT-qPCR can complement protein analyses; for example, elevated c-FLIP mRNA in cervical intraepithelial neoplasia/cervical cancer associates with disease progression, although diagnostic sensitivity remains uncertain [303]. Flow cytometry–based assays have been developed to quantify c-FLIP protein in circulating CD14⁺ monocytes from patient blood, expanding biomarker modalities [177]. Continued maturation of single-cell and spatial omics should resolve c-FLIP heterogeneity across cell types, disease states, and niches, enabling isoform-aware and context-specific targeting. Advances in detection will refine patient stratification and pharmacodynamic monitoring.

### Immune-associated diseases: autoimmunity, inflammatory disorders, and allergy

Immune dysregulation commonly manifests as allergy and autoimmunity, reflecting maladaptive activation of protective pathways [304]. Classic Gell–Coombs hypersensitivity types (I–IV) categorize these processes by mechanism: IgE-mediated immediate reactions (type I), antibody-dependent cytotoxicity (type II), immune complex–driven injury (type III), and T cell–mediated delayed responses (type IV) [305]. In type I (allergic) disease, allergen-specific IgE engages FcεRI on mast cells and basophils, triggering degranulation (histamine release) and lipid mediator release (leukotrienes) that drive immediate and late-phase responses [306]. Type II disease results from IgG/IgM binding to surface antigens, activating complement and cytotoxicity [305]. Type III injury derives from deposition of antigen–antibody complexes with Fc receptor/complement activation [305]. Type IV reflects antigen-specific T-cell responses with delayed, localized inflammation and, in some contexts, granuloma formation [305]. Breakdown of self-tolerance via failed deletion or control of autoreactive clones underlies autoimmune phenotypes, causing local or systemic inflammation and tissue damage [307]. A unifying theme across these states is defective control of cell death and survival, in which c-FLIP plays a central role by perturbing tolerance, fuelling chronic inflammation, and shaping tissue pathology. Below, we summarize evidence linking c-FLIP to hypersensitivity and autoimmune diseases and highlight therapeutic implications.

### Type I disorders

#### Asthma

Asthma is a chronic inflammatory disease of the lower airways characterized by variable airflow limitation and bronchial hyper-responsiveness to irritants and allergens; genetics and environment both contribute [308, 309]. c-FLIP appears to regulate apoptosis and inflammatory tone in airway tissues. In severe, corticosteroid-resistant patients, both c-FLIP_L_ and c-FLIP_S_ are reduced in PBMCs compared with controls, implicating dysregulation of extrinsic apoptosis in refractory disease [310]. Lower FLIP may amplify inflammatory cell death and release of DAMPs, worsening local inflammation. In bronchial epithelium, *CFLAR* is decreased in smokers; *CFLAR* knockdown accelerates epithelial necrosis and DAMP release, suggesting that c-FLIP loss promotes epithelial injury and chronic airway inflammation [311]. At the T-cell level, enhanced c-FLIP_L_ in activated CD4⁺ cells promotes Th2 cytokine production and suppresses Th1 cytokines, thereby exacerbating allergic inflammation beyond its canonical anti-apoptotic role [21]. Among effector granulocytes, TNF-α induces c-FLIP_L_ in eosinophils, supporting their survival in vitro and in vivo [312]. Collectively, cell–type–specific changes in c-FLIP shape epithelial integrity, Th2 skewing, and eosinophil persistence in asthma.

#### Inflammatory skin diseases

FADD–caspase-8–c-FLIP signaling tightly regulates keratinocyte survival. Indeed, c-FLIP is highly expressed in basal epidermis. Complete prenatal epidermal *Cflar* deletion is lethal, whereas acute postnatal deletion in the K14CreER^tam^ transgenic mouse strain disrupts epidermal homeostasis, underscoring its non-redundant role [313]. Human dyskeratosis shows reduced total c-FLIP protein expression and TNF-dependent keratinocyte apoptosis; TNF blockade mitigates early inflammation and cell death, consistent with a protective role of c-FLIP against TNF-mediated injury. Loss of epidermal c-FLIP may be a prerequisite for catastrophic keratinocyte death in toxic epidermal necrolysis (TEN)/Stevens-Johnson syndrome (SJS) or erythema exudativum multiforme (EEM) [313]. The crucial role of c-FLIP for epidermal integrity and skin inflammation silencing has been more recently confirmed in c-FLIP epidermal KO mice with the concomitant TNF deficiency [314]. Nevertheless, further investigations are essential to dissect the role of specific isoforms in human dyskeratosis.

### Type II autoimmune diseases

#### Graves’ disease (GD) and Hashimoto’s thyroiditis (HT)

GD, a leading cause of hyperthyroidism, reflects stimulatory TSHR autoantibodies (TRAb) that drive hormone excess and extrathyroidal manifestations (orbitopathy, dermopathy) with significant morbidity [315, 316]. HT features thyrocyte destruction mediated by autoreactive T cells and thyroid-specific autoantibodies, and is a major cause of hypothyroidism, especially in women; genetic and environmental factors contribute [317, 318]. Histologically, thyroid autoimmunity shows lymphocytic infiltration, but outcomes diverge: thyrocyte loss in HT versus thyrotoxicosis with viable thyrocytes in GD [319, 320]. Mechanistically, HT thyrocytes upregulate FasR and FasL and are sensitive to CD95-mediated apoptosis, amplified by Th1 cytokines (IFN-γ) that increase caspases [321]. In contrast, GD thyrocytes exhibit weaker FasR yet express FasL and resist apoptosis owing to Th2 cytokine–driven upregulation of c-FLIP_L_ and Bcl-xL [320, 321]. Thus, Th1/Th2 cytokine milieus differentially program apoptotic sensitivity via c-FLIP. Preclinical models reinforce context dependence. In spontaneous autoimmune thyroiditis (SAT), infiltrating leukocytes express high levels of c-FLIP_L_, FasR, and FasL; c-FLIP blocks Fas-mediated apoptosis of inflammatory cells, thereby promoting chronicity. In contrast, in granulomatous experimental autoimmune thyroiditis (G-EAT), thyrocytes express c-FLIP_L_ and FasL with increased active caspase-8/-3, favoring apoptosis of infiltrating cells and resolution. Hence, c-FLIP_L_ in inflammatory cells supports persistence, whereas c-FLIP in thyrocytes aligns with disease resolution [322].

#### Myasthenia Gravis (MG)

MG is a prototypic antibody-mediated neuromuscular disease caused by autoantibodies targeting acetylcholine-receptor (AChR) or muscle-specific kinase (MuSK) epitopes at the postsynaptic membrane. In approximately 15% of patients, MG is associated with thymoma, a neoplasm that disrupts thymic selection and tolerance. Within this setting, T cells display marked up-regulation of c-FLIP_L_ in both thymic tissue and circulating peripheral blood mononuclear cells. Elevated c-FLIP_L_ expression renders thymic and peripheral T cells resistant to FasL-induced apoptosis, thereby impairing clonal deletion of autoreactive clones. Mechanistically, c-FLIP_L_ overexpression in thymoma-associated MG correlates with increased NF-κB activation, suggesting that oncogenic or inflammatory signals within the tumor microenvironment stabilize c-FLIP transcription and protein turnover [323]. Following thymectomy, c-FLIP_L_ expression in peripheral lymphocytes declines, paralleling clinical improvement. Although direct therapeutic manipulation of c-FLIP in MG has not yet been tested, normalization of its expression after thymectomy and immunosuppression suggests that targeting NF-κB–c-FLIP signaling could complement conventional therapies aimed at restoring tolerance. Unfortunately, the assessment of c-FLIP protein expression in this disease context has been investigated using an anti-c-FLIP antibody that recognizes only the long isoform of the protein. Therefore, the variation and the potential contribution of the short c-FLIP isoforms are still lacking.

### Type III immune-complex disease

#### Systemic lupus erythematosus (SLE)

SLE involves pathogenic autoantibodies to nuclear antigens, immune complex deposition, and multi-organ injury with a relapsing–remitting course. Unlike normal T cells that downregulate c-FLIP (notably c-FLIP_S_), SLE T cells upregulate anti-apoptotic molecules, including COX-2, c-FLIP_L_ and c-FLIP_S_, Mcl-1, and c-IAPs, promoting anergy resistance and apoptosis evasion. COX-2 inhibitors (celecoxib, niflumic acid, SC58125) restore apoptosis of anergy-resistant lupus T cells by reducing anti-apoptotic proteins; celecoxib and niflumic acid particularly downregulate both c-FLIP_L_ and c-FLIP_S_, whereas Bcl-2 family members are largely unaffected [324]. This supports a COX-2/c-FLIP, PGE2-independent survival program in lupus T cells, distinct from COX-2–Bcl-2/Akt/Mcl-1 axes in cancer. Genetically, constitutive expression of the murine short splice variant c-FLIP_R_ (VavFLIPR mouse model) protects lymphocytes from CD95- and activation-induced cell death; with age, mice develop activated B/T phenotypes, anti-nuclear antibodies, and kidney/lung pathology reminiscent of SLE, indicating that enforced short-isoform c-FLIP can drive autoimmunity [22].

### Type IV T-cell–mediated diseases

#### Periodontitis

Periodontitis is a prevalent chronic inflammatory disease (severe forms ~11% globally) driven by dysbiotic Gram-negative subgingival biofilms and maladaptive host responses, culminating in destruction of the periodontal ligament and alveolar bone [325]. Periodontopathogens induce inflammatory cytokines (e.g., TNF-α) and multiple regulated cell-death programs (apoptosis, necroptosis, pyroptosis, NETosis). Butyric acid, an abundant microbial metabolite, induces apoptosis in human T cells; TNF-α augments this effect by enhancing caspase activity. Mechanistically, butyrate downregulates c-FLIP in a dose-dependent manner, increasing TNF-α sensitivity through DR–caspase signaling [326]. *Porphyromonas gingivalis* activates RIPK1/RIPK3/MLKL-dependent necroptosis and inflammatory gene programs in monocytes. Inhibition or knockdown of CDK9 suppresses inflammatory responses, increases caspase-8, and decreases c-FLIP, RIPK1, RIPK3, and TRIF, thereby attenuating CDK9–caspase-8–c-FLIP-dependent necroptosis—implicating an axis in periodontal inflammation [327].

#### Inflammatory bowel diseases (IBD)

IBDs, including Crohn’s disease (CD) and ulcerative colitis (UC), are relapsing inflammatory disorders of the gastrointestinal (GI) tract with rising incidence and increased CRC risk; environmental changes (e.g., diet) likely modulate disease expression [328, 329]. Excessive necroptosis disrupts epithelial barrier integrity and fuels inflammation. During necroptosis, phospho-MLKL can translocate to mitochondria, provoking microtubule-dependent mitochondrial DNA release that activates cGAS–STING and IFN-β expression; cGAS–STING inhibition reduces inflammation and aids recovery in murine colitis [330]. c-FLIP_L_ restrains assembly of the “Ripoptosome” in intestinal epithelial cells and functions as a key antinecroptotic factor; thus, loss or reduction of c-FLIP may permit MLKL activation, mtDNA release, and cGAS–STING–driven inflammation. In Crohn’s disease (CD), lamina propria T lymphocytes (LPLs) are resistant to Fas-mediated apoptosis, and this defect contributes to the mucosal T-cell accumulation. After Fas stimulation, the rate of apoptosis of CD3^+^ LPLs was higher in normal controls and patients with UC than in CD patients, and an antisense oligonucleotide-mediated c-FLIP inhibition was able to reverse the resistance of CD LPLs to Fas-induced apoptosis. Furthermore, enhanced expression of both long and short c-FLIP isoforms was reported in biopsy specimens and purified CD3^+^CD45RO^+^ LPLs from CD patients in comparison with UC patients and normal controls [331]. These data clearly suggest a role of c-FLIP in the resistance of LPLs to Fas-mediated apoptosis, especially in CD patients. Notably, c-FLIP overexpression was not observed in celiac disease mucosa, suggesting that induction of c-FLIP in the gut may not simply rely on ongoing inflammation [332].

#### Psoriasis

Psoriasis is a chronic papulosquamous disease ( > 60 million worldwide) with epidermal hyperplasia, IL-23/Th17-driven inflammation, and notable comorbidities [333, 334]. Autoreactivity to antigens, such as the melanocyte antigen ADAMTSL5, the cathelicidin antimicrobial peptides LL37 or KRT17, by tissue-resident memory T cells sustains lesions [334]. Keratinocytes in psoriatic skin exhibit cytokine-induced hyperproliferation/differentiation abnormalities and resist apoptosis. c-FLIP (mRNA/protein) and Bcl-xL are increased in psoriatic keratinocytes compared with normal epidermis, correlating with apoptosis resistance to IFN-γ/TNF-α [335, 336]. Therapies that restore apoptosis (e.g., TNF antagonists, methotrexate) reduce expression of Bcl-xL, c-FLIP, NF-κB p65, and pAkt1 and increase caspase-9, aligning clinical improvement with recalibrated death/survival programs.

#### Rheumatoid arthritis (RA)

RA is a common chronic systemic autoimmune disease marked by symmetric inflammatory polyarthritis, synovial hyperplasia, and progressive bone/cartilage destruction; prevalence is ~0.5–1.0%, with female predominance and genetic–environmental interplay [337]. HLA-DR “shared epitope” alleles dominate genetic risk; additional loci include PTPN22 (gain-of-function), IL-6R coding variation, and regions near TRAF1–C5 [337]. TRAF-1, an adapter for TNFR family signaling, both promotes NF-κB/MAPK downstream of TNFR1/2 (via cellular inhibitor of apoptosis 2, cIAP2) and restrains TLR pathways; TRAF1-deficient mice exhibit exaggerated T-cell proliferation to TNF and anti-CD3, indicating a negative regulatory role [338]. Defective apoptosis contributes to synovitis. In early RA, synovial macrophages and c-FLIP expression are high with minimal apoptosis; in longstanding RA, macrophage numbers and c-FLIP total proteins fall while apoptosis rises (8.8% vs 0.6% in early disease) [339]. c-FLIP is absent from normal synovium but enriched in RA. Interestingly, both CD68-positive macrophage-like cells and CD68-negative fibroblast-like cells expressed c-FLIP_L_. This protein was primarily localized in areas where tissue invaded cartilage and bone, as well as in the synovial lining. Additionally, at sites of matrix degradation, multinucleated cells showed positive staining for c-FLIP_L_, suggesting a prolonged survival of cells that secrete matrix metalloproteinases and cathepsins [340]. Finally, mice with conditional deletion of c-FLIP in CD11c^+^ cells spontaneously develop a syndrome similar to RA, characterized by erosive, inflammatory arthritis, the presence of autoreactive CD4^+^ T cells, and autoantibodies specific for joint tissue [23], exploring how the absence of c-FLIP during DC differentiation may influence the development of natural Tregs in the thymus.

#### Multiple sclerosis (MS)

MS is a chronic T cell–mediated demyelinating disease of the central nervous system (CNS) with rising global prevalence and substantial disability burden [341, 342]. Activated T cells from relapsing–remitting MS (RR-MS) patients overexpress both c-FLIP_L_ and c-FLIP_S_, and resist FasR-mediated apoptosis relative to controls, identifying anti-apoptotic c-FLIP upregulation as a feature of clinical activity [343]. Together with adaptive immune cells, innate cell components, such as macrophages and microglia, have been shown in numerous studies to play crucial roles in the onset and progression of MS. Microglia, the resident phagocytic cells of the CNS, are abundant in lesions associated with MS, as observed in both autopsy specimens and the experimental autoimmune encephalomyelitis (EAE) model [343]. Inflammasome activation (e.g., NLRP3) is a key pathogenic driver. *Porphyromonas gingivalis*, a periodontitis pathogen, enhances macrophage inflammasome activation through the TLR4–TRIF–MyD88–HIF-1α axis under hypoxia; infection worsens EAE in a HIF-1α–dependent manner, linking mucosal pathogens to CNS inflammation [344]. Mechanistically, c-FLIP_L_ associates with NLRP3 and supports optimal NLRP3 and AIM2 inflammasome assembly, potentially via inhibition of autophagy, suggesting c-FLIP_L_ as a target in inflammasome-overactivation disorders [112]. Conversely, in oligodendrocytes, c-FLIP_L_ is protective. IFN-γ can prevent TNF-α–induced apoptosis in committed oligodendrocytes via NF-κB–dependent induction of c-FLIP_L_ and JAK-mediated PKR activation. Gain/loss studies and an IL-1βXAT model of sustained CNS inflammation show that c-FLIP_L_ upregulation is necessary and sufficient for oligodendrocyte survival; c-FLIP knockdown in vivo increases oligodendrocyte death despite inflammation, highlighting cell–type–specific benefits of c-FLIP_L_ in demyelinating settings [345].

### Other pathologies

c-FLIP is now recognized as a context-dependent rheostat that integrates death-receptor inputs (FasR/TNFR/TRAIL-R) with kinase, transcriptional, metabolic, and stress pathways to determine whether cells execute apoptosis or survive, and whether tissues repair or remodel pathologically. Below, we synthesize evidence implicating c-FLIP in cardiovascular and metabolic disease, chronic lung disorders, brain injury and neurodegeneration, kidney disease, COVID-19, and sepsis, highlighting common mechanistic themes and pointing to therapeutic opportunities.

#### Cardiovascular diseases

The World Health Organization (WHO) reported that cardiovascular diseases are the leading cause of death globally, accounting for approximately 38% of all premature deaths from noncommunicable diseases [346]. Within the myocardium, c-FLIP influences outcomes not only by tempering apoptosis but also by shaping the quality of stress adaptation and remodeling. In ventricular myocardium from patients with end-stage heart failure and in mouse hearts after myocardial infarction (MI), c-FLIP_L_ expression is significantly reduced [347, 348]. Genetic ablation underscores developmental and structural requirements: c-FLIP knockout mice display impaired trabeculation and a thinned myocardium, indicating a fundamental role in cardiac morphogenesis and myocardial integrity [349]. Cardiac stressors such as pressure overload or neurohormonal drive (e.g., angiotensin II) commonly downregulate c-FLIP_L_; this correlates with aggravated hypertrophy, interstitial fibrosis, and declining ventricular performance [350]. Mechanistically, low c-FLIP permits unchecked activation of pro-apoptotic and pro-fibrotic signaling nodes, such as ER stress and p38 MAPK, hereby exacerbating adverse remodeling [351]. In contrast, cardiac-restricted c-FLIP overexpression mitigates pressure-overload hypertrophy and preserves function, in association with suppression of Akt/GSK3β and ERK1/2 signaling axes [154]. Thus, c-FLIP acts as a molecular “brake” that curbs both myocyte death and maladaptive growth programs during hemodynamic challenge.

Atherosclerotic coronary heart disease (CHD), the most prevalent cardiovascular condition, emerges from a chronic inflammatory response to subendothelial lipids, involving endothelial cells (ECs), vascular smooth muscle cells (SMCs), and myeloid cells [352, 353]. In non-atherosclerotic human coronary arteries, c-FLIP is abundant and is associated with a low apoptotic index; within plaques, total c-FLIP protein is reduced or spatially heterogeneous, particularly in ECs and SMCs, and this is associated with increased TUNEL staining [354]. These observations suggest that c-FLIP loss favors cell death within plaques, potentially undermining cap stability and accelerating progression to clinically dangerous states (plaque narrowing, rupture, thrombosis) [352–354]. Collectively, these cardiac and vascular data position c-FLIP, especially the long isoform, as a key determinant of cell viability and structural remodeling in cardiovascular disease, with implications for heart failure, ischemic injury, and plaque stability.

#### Metabolic disorders

Metabolic syndrome and its components, including type 2 diabetes (T2D), obesity, and metabolic dysfunction-associated fatty liver disease (MAFLD), have risen sharply, affecting ~20–25% of adults and projecting steep increases in disease burden in the coming decades [355–357]. c-FLIP is integral to how metabolic tissues respond to glucolipotoxic and inflammatory stress.

In human islets, c-FLIP is normally expressed and protects β cells from apoptosis elicited by high glucose and inflammatory cytokines [358]. In T2D, β-cell c-FLIP levels decline; exposure of human islets to hyperglycemia lowers both c-FLIP_L_ and c-FLIP_S_ and increases apoptosis. Conversely, augmenting c-FLIP redirects FasR signals away from death toward survival, restores β-cell proliferation, and improves function [358]. The link to glucotoxicity is strengthened by the finding that high glucose induces β-cell FasR (with neighboring β-cells providing constitutive FasL), thereby priming Fas-mediated apoptosis and accelerating β-cell attrition in T2D [359]. In type 1 diabetes models, enforced c-FLIP prevents cytokine-triggered caspase-8 activation, sustains NF-κB–dependent survival programs, and preserves insulin secretion responses that likely aid β-cells facing inflammatory and metabolic stress in insulin-resistant states [360]. Together, these data place c-FLIP at a pivotal checkpoint for β-cell fate under diabetogenic conditions.

Obesity and sedentary lifestyle fuel metabolic syndrome and MAFLD progression [361, 362]. In high-fat diet (HFD) stress, JNK activation stimulates the E3 ligase ITCH, promoting proteasomal degradation of c-FLIP and rendering hepatocytes vulnerable to apoptosis and necroptosis—thereby intensifying liver injury, inflammation, and evolution toward steatohepatitis [363]. In parallel, proinflammatory ω-6–driven cues activate NF-κB and can transcriptionally upregulate c-FLIP as a compensatory cytoprotective response; yet chronic metabolic stress often overwhelms this buffer, culminating in hepatocyte death and inflammatory amplification [364, 365]. Hepatocyte-specific c-FLIP deletion confirms its protective role by heightening death receptor–mediated apoptosis and liver injury in vivo [366, 367].

MAFLD spans simple steatosis to metabolic dysfunction-associated steatohepatitis (MASH) [368]. MASH progression features hepatocyte death tightly linked to FasR, TNFR1, and TRAIL-R pathways. In this context, c-FLIP functions as an essential inhibitor of caspase-8 and, thereby, of apoptosis [369, 370]. Diminished c-FLIP facilitates death-receptor killing, driving injury, inflammation, and fibrosis; maintaining c-FLIP counters lipotoxic apoptosis and blunts disease progression [371]. Consistent with this, hepatic neuregulin-4 (Nrg4) signaling prevents ubiquitination and proteasomal degradation of c-FLIP_L_, thereby stabilizing its cytoprotective functions and attenuating the transition from steatosis to MASH [372]. These converging lines of evidence identify c-FLIP_L_ as a linchpin in hepatocyte survival during metabolic stress.

#### Lung diseases

Idiopathic pulmonary fibrosis (IPF) and chronic obstructive pulmonary disease (COPD) are both chronic lung diseases that cause progressive respiratory impairment but differ fundamentally in their pathology and clinical features [373]. IPF is a relentlessly progressive fibrosing interstitial pneumonia with limited therapeutic options and a median survival of only a few years after diagnosis [374, 375]. In IPF lungs, total c-FLIP protein is distinctly upregulated in alveolar epithelial cells at injury/fibrosis interfaces [376], and overexpressed in myofibroblasts in bleomycin-challenged mice and in human disease [377]. Functionally, enhanced c-FLIP confers resistance to Fas-mediated apoptosis in fibrotic myofibroblasts and can even bias FasR signaling toward proliferation, whereas epithelial c-FLIP supports the survival of cells undergoing epithelial–mesenchymal transition (EMT). This compartmentalized pattern of overexpression between epithelial and stromal cells tilts the balance toward persistence of apoptosis-resistant, matrix-producing cells and progressive scarring [377]. These observations place c-FLIP at the intersection of epithelial injury responses and fibroproliferative remodeling in IPF [377].

COPD is a common, preventable, and treatable disease characterized by persistent airflow limitation and chronic airway/alveolar inflammation, most often driven by long-term exposure to cigarette smoke [378–380]. Emerging data implicate c-FLIP as a modulator of disease evolution by altering the balance between apoptosis and necroptosis in airway epithelial and immune cells [381]. c-FLIP normally restrains caspase-8; when perturbed (e.g., by smoke), cells may default to necroptosis, a DAMP-releasing, proinflammatory death mode that sustains airway inflammation and tissue damage. Preliminary evidence suggests that dysregulated c-FLIP contributes to chronic inflammatory loops and structural decline in COPD, proposing c-FLIP as both a biomarker and a potential therapeutic lever [381].

#### Brain diseases

c-FLIP is expressed in the brain and plays a significant role in regulating programmed cell death pathways, particularly after brain injury and in Alzheimer’s disease (AD) [70, 382]. c-FLIP is expressed in the adult brain and dynamically regulated after injury. In mice, Hainsworth et al. demonstrated that the c-FLIP_R_ isoform, that is the only murine short FLIP isoform reported to date, predominates at baseline, with lower c-FLIP_L_ levels; following controlled cortical impact, c-FLIP_L_ rises early, dips by ~12 h, then re-accumulates between 24–72 h, whereas c-FLIP_R_ falls in dying cortical neurons but persists in hippocampal neurons [70]. Reactive astrocytes near lesion sites later upregulate c-FLIP_R_, possibly supporting astroglial survival and repair. In this study, the authors used a rarely used nomenclature for FLIP isoforms, classifying the short c-FLIP isoforms as c-FLIP-δ. Nevertheless, the modern nomenclature consistently defines the murine short c-FLIP isoform as c-FLIP_R_. Human data mirror these compensations: c-FLIP_L_ is elevated in severe traumatic brain injury (TBI) specimens compared with epilepsy controls, consistent with an endogenous protective response [383]. Beyond apoptosis, c-FLIP can modulate pyroptosis in neurons by regulating GSDMD cleavage, thereby linking *CFLAR* to inflammatory cell death circuits relevant to neurodegeneration [114]. Collectively, these kinetic and spatial patterns argue that a finely regulated balance of c-FLIP isoforms is a key determinant of neuronal and glial survival during the primary and secondary injury phases.

AD, the most common cause of dementia, is increasing rapidly with population aging [384]. Hallmark pathology includes extracellular Aβ plaques, intracellular tau tangles, chronic glial activation, synaptic failure, and mitochondrial/oxidative stress [384, 385]. c-FLIP regulation in AD appears heterogeneous and cell-type specific. In advanced AD, brain regions enriched for hyperphosphorylated tau exhibit decreased miR-512 with concomitant neuronal upregulation of total c-FLIP protein and MCL1, which are direct miR-512 targets. This shift may stabilize dysfunctional neurons by suppressing apoptosis, potentially prolonging the persistence of pathology and contributing to clinical decline [386]. In contrast, several vascular and parenchymal compartments show c-FLIP reduction: brain microvascular ECs in AD downregulate *CFLAR*, which may amplify pro-apoptotic signaling, promote pericyte loss, and compromise blood–brain barrier integrity [387]. Aβ oligomers activate DR4/DR5 on brain ECs while diminishing c-FLIP expression/activity, thus sensitizing the endothelium to apoptosis and vascular dysfunction [382]. Broad tissue analyses report reduced c-FLIP_L_ protein in the frontal cortex and cerebellum, with reciprocal changes in ARC and RIPK2, suggesting complex re-wiring of death networks that may paradoxically inhibit classical apoptosis while sustaining dysfunctional neuronal survival and inflammation [388]. Finally, hippocampal samples from AD brains show signatures of necroptosis, as evidenced by elevated and phosphorylated RIPK3/MLKL, along with downregulated *CFLAR* and reduced NF-κB activity, supporting a shift from apoptosis to necroptosis as the dominant death program [389]. Together, these findings place c-FLIP at the nexus of neuronal, glial, and vascular stress responses along the AD continuum.

#### Kidney diseases

Chronic kidney disease (CKD), acute kidney injury (AKI), and polycystic kidney disease (PKD) primarily result from an imbalance between the death of parenchymal cells and the proliferation or recruitment of maladaptive cells. These renal conditions are among the fastest-growing causes of mortality and may rank fifth worldwide by 2040 [390].

In ischaemia–reperfusion injury-induced AKI (IRI-AKI), tubular epithelial apoptosis accelerates as c-Myc becomes markedly induced and transcriptionally represses c-FLIP by promoter binding. The resulting decline in c-FLIP_L_ facilitates caspase-8 and caspase-3 activation via FasL/FasR, thereby worsening tubular loss [2]. Overexpressing c-FLIP in renal epithelia attenuates FasL-driven apoptosis; pharmaco-genetic c-Myc inhibition restores c-FLIP, reduces caspase activation, and ameliorates tubular injury in experimental models [2]. These data place c-FLIP downstream of stress-induced c-Myc as a pivotal guardian of tubular survival during AKI.

In healthy kidneys, c-FLIP_L_ and p43-FLIP are expressed in cortex and tubular epithelial cells (TECs), where they restrain caspase-8 and limit apoptosis—functions essential for epithelial integrity [391, 392]. Inflammatory renal milieus (autoimmune nephritis, allograft rejection) can downregulate TEC c-FLIP_L_ (e.g., via IL-2), thereby enhancing apoptosis and promoting tubular atrophy and interstitial inflammation [391]. Conversely, TEC c-FLIP_L_ and p43-FLIP overexpression attenuates cytokine-induced death. c-FLIP different isoforms and their cleavage products also intersect NF-κB-dependent programs that influence survival and inflammatory tone during CKD evolution [393, 394]. These context-specific effects make c-FLIP a potential target to reduce tubular dropout and slow CKD progression.

In PKD (including autosomal dominant forms), c-FLIP_L_ is upregulated in cystic epithelia and in kidneys from Pkd1 mutant mice, and TNF-α present in cyst fluid can induce its expression [395–397]. Elevated c-FLIP suppresses TNFR1-mediated extrinsic apoptosis, facilitating persistence and growth of cystic epithelia; in large human cysts, c-FLIP abundance inversely correlates with caspase activation [397]. Thus, c-FLIP-dependent evasion of apoptosis appears to support cyst expansion and progressive kidney dysfunction [395–397].

#### Coronavirus disease 2019 (COVID-19)

Severe COVID-19 is characterized by dysregulated innate immunity (impaired interferon signaling, hyperactive NF-κB), culminating in a cytokine storm, endothelial damage, and multiorgan failure [398–402]. Early after SARS-CoV-2 infection, c-FLIP isoforms are upregulated in lung epithelial cells and circulating myeloid populations from patients [403]. By inhibiting caspase-8, c-FLIP delays apoptosis of infected cells, potentially prolonging viral production. In myeloid cells, total c-FLIP protein correlates with STAT3 activation and features of cytokine release syndromes, implicating a c-FLIP–STAT3 axis in the hyperinflammatory phenotype of severe disease [404]. Preclinical interference with this axis reduces inflammation and attenuates disease severity, highlighting c-FLIP as a candidate therapeutic node that links apoptosis restraint to immunopathology in COVID-19.

#### Sepsis

Sepsis and septic shock remain major global killers, with ~48.9 million cases and ~11.0 million deaths in 2017—nearly one-fifth of worldwide mortality [405]. The syndrome features an early hyperinflammatory wave followed by sustained immunosuppression, with multiorgan dysfunction across cardiovascular, coagulation, pulmonary, hepatic, renal, and CNS systems [406–408]. In this context, c-FLIP shapes both endothelial viability and immune responses. In human umbilical vein endothelial cells (HUVECs), c-FLIP overexpression protects against apoptosis induced by LPS and cycloheximide; complement C5a aggravates injury by enhancing caspase-8 activation and suppressing c-FLIP [409]. In vivo, c-FLIP_L_ declines across organs after cecal ligation and puncture (CLP) sepsis, paralleling vascular dysfunction and disease progression [409]. Inducible c-FLIP knockout precipitates acute liver failure with hypoglycemia, hyperbilirubinemia, and systemic inflammation (TNF-α, IL-6), preceded by accumulation of proinflammatory M1 macrophages and demonstrating a requirement for c-FLIP in maintaining hepatic immune homeostasis under systemic stress [410]. Clinically, *CFLAR* is part of a four-gene prognostic signature (*CFLAR*, *CXCL8*, *FASLG*, *TP53*) that stratifies sepsis survival and corresponds to distinctive immune-cell infiltration patterns, supporting both the biomarker’s value and its mechanistic relevance [411]. Moreover, heightened glucose-metabolism scores in monocytes and NK cells in sepsis associate with CFLAR-linked pathways, tying c-FLIP to immune metabolic reprogramming in critical illness [412].

### c-FLIP as a potential therapeutic target

On the therapeutic front, diversified approaches, including chemotherapy-induced downregulation, RNAi/LNP delivery, small-molecule disruptors of DISC recruitment or caspase-8/c-FLIP complexes, proteostasis-based degradation, and metabolic epigenetic reprogramming, are converging to render c-FLIP druggable. Strategic combinations that integrate these modalities with DR agonists or immunotherapies hold promise for restoring apoptotic competence and improving outcomes, especially in c-FLIP–addicted cancers (Table 2). Continued research into the regulation and function of c-FLIP is essential for the rational design of targeted therapies to restore apoptosis and improve treatment outcomes. Here, we report potential therapeutic approaches to interfere with c-FLIP expression and function, classified by their mechanisms of action.

**Table 2:** Summary of Targeting c-FLIP for the Treatment of Human Diseases.

Drug type	Targeting approach	Mechanism of action	Representative references
*Direct targeting*			
Gene Silencing	siRNA / shRNA	Knockdown of c-FLIP mRNA results in reduced protein expression	[417, 451]
	Antisense oligonucleotides (ASOs)	Target the AUG translational initiation sequence of c-FLIP to reduce protein translation	[452]
Small Molecule	FLIPin family (e.g., FLIPinB)	Disrupts c-FLIP binding to FADD/caspase-8 in the DISC	[453]
	c-FLIP inhibitors	Disrupt c-FLIP/caspase-8 heterodimer formation via DED2 domain	[418]
Natural compounds	Lupeol	Binds to the p20 segment of c-FLIPL, preventing its dimerization with caspase-8	[454, 455]
Combination Therapy	Vorinostat + c-FLIP siRNA	Transcriptional and post-transcriptional inhibition of c-FLIP	[416]
*Indirect Targeting*			
Proteasomal degradation	SAHA (vorinostat)	Induces c-FLIP(L/S) degradation via ubiquitin-proteasome pathway	[59]
	PPARγ agonists	Promote c-FLIP ubiquitination and proteasomal degradation	[456]
	Tetrahydrocurcumin (THC)	Targets TRIPK13 to disrupt TRIPK13/USP7/c-FLIP complex, promoting c-FLIP ubiquitination	[457]
	Quercetin	Activates JNK in a dose-dependent manner, leading to proteasomal degradation of c-FLIP	[458]
RNA Stability	DZNep (3-Deazaneplanocin A)	Reduces c-FLIP mRNA and protein stability	[420]
	Maritoclax	Upregulates miR-708 expression, which suppresses c-FLIP translation or promotes cFLIP mRNA degradation	[459]
	HuR inhibitor (KH-3)	Inhibits RNA-binding protein HuR, destabilizing c-FLIP mRNA	[460]
Epigenetic Regulation/ Metabolic modulation	HDAC inhibitors (e.g., MS275)	Induce nuclear c-Myc, which binds the c-FLIP promoter and reduces its transcription	[261]
	Glucose deprivation	Decreases superoxide levels, downregulating c-FLIP expression	[293]
	Inhibition of glutaminolysis	Reduces α-KG production and increases the H3K9me3 histone mark at the c-FLIP promoter	[61]
Transcriptional downregulation	MCPIP1 (Monocyte chemoattractant protein-1-induced protein 1)	Inhibition of NF-κB activation, reducing c-FLIP transcription	[461]
Unspecified	Dinaciclib (CDK9 inhibitor)	Unspecified mechanism	[462]

#### Chemotherapy-mediated downregulation

Conventional agents, such as cisplatin, 5-fluorouracil, gemcitabine, etoposide, and paclitaxel, can lower c-FLIP levels across tumor models, thereby reducing the apoptotic threshold [54, 241, 248, 413–415]. Mechanisms vary by context (transcriptional repression, translational control, enhanced proteasomal turnover), and schedule-dependent combinations with death-ligands or DR agonists may be required for durable responses [3]. These findings support the concept that transient c-FLIP suppression can prime tumor cells for extrinsic apoptosis.

#### RNA-based therapeutic approaches

Advances in delivery platforms have revitalized nucleic acid therapeutics targeting c-FLIP. Multifunctional nanoparticles can deliver c-FLIP–specific siRNA to tumors, leading to robust knockdown and growth inhibition in preclinical models [416]. shRNA targeting of c-FLIP sensitizes cervical adenocarcinoma to chemo-radiotherapy [417]. With clinical progress in lipid nanoparticles (LNPs) for mRNA delivery, analogous vehicles for siRNA/antisense against c-FLIP are increasingly feasible. These approaches directly reduce c-FLIP mRNA abundance and may offer high specificity when combined with a tumor-targeted delivery system.

#### Small-molecule mechanistically interfering with c-FLIP function

Developing small-molecule inhibitors targeting c-FLIP remains challenging due to its structural homology with caspase-8 [10]. Nonetheless, “FLIP-in” compounds that bind c-FLIP within the caspase-8/c-FLIP heterodimer have been reported to enhance caspase-8 catalytic activity and restore apoptosis [418]. High-throughput and in silico screenings are beginning to yield candidates that engage c-FLIP’s DED2 domain; by blocking c-FLIP recruitment to the DISC without impairing pro-caspase-8 docking, these agents re-sensitize cells to TRAIL [418].

#### Destabilizing c-FLIP at the mRNA/protein level

Targeting c-FLIP degradation is also a potential direction for developing innovative therapeutic strategies. An orthogonal tactic is to accelerate c-FLIP turnover. Withanolide E in renal cancer enhances c-FLIP degradation, rapidly lowering protein levels and sensitizing cells to TRAIL [419]. In B-cell lymphoma, the pan-methyltransferase inhibitor DZNep accelerates c-FLIP loss and promotes caspase-8 processing, potentially via upregulation of microRNAs that destabilize c-FLIP mRNA [420]. The pro-apoptotic serine protease HtrA2 can directly degrade c-FLIP; pharmacologic activation of HtrA2 may re-enable DISC signaling [421].

#### Metabolic/epigenetic modulation

Metabolic epigenetics also regulates c-FLIP expression: in pancreatic cancer, glutamine-derived α-ketoglutarate (α-KG) fuels KDM4C-dependent histone demethylation at the c-FLIP promoter, increasing transcription. Inhibiting glutaminolysis lowers α-KG, attenuates KDM4C activity, downregulates c-FLIP, and restores TRAIL sensitivity [61]. Direct chromatin modification agents also have the potential to regulate c-FLIP expression. HDAC inhibitors promote the nuclear transport of c-Myc, which binds to the promoter region of *CFLAR* and represses its transcription, ultimately reducing cytoplasmic c-FLIP level [61].

## Conclusion

The regulatory function of c-FLIP at the DISC and dynamic changes in c-FLIP levels critically determine the balance between cytoprotection and cell elimination. The biological impact of c-FLIP in disease conditions is highly dependent on cellular lineage, isoform expression, and the temporal stage of disease. Upregulation of c-FLIP may be cytoprotective in specific cell types, whereas its reduction could facilitate the clearance of apoptosis-resistant cells in particular pathological conditions. Thus, the therapeutic modulation of c-FLIP requires precise spatiotemporal and isoform-specific targeting.

Multiple regulatory tiers govern c-FLIP expression. Transcriptionally, it is induced by NF-κB signaling during inflammation and repressed by c-Myc under stress conditions such as AKI [2, 56, 132, 133, 261]. Post-translationally, its turnover is driven by JNK-ITCH–mediated ubiquitination and proteasomal degradation, notably in metabolic liver injury [19, 59, 73, 142, 422]. Signal integration through Akt/GSK3β, ERK1/2, p38 MAPK, and ER stress further shapes c-FLIP abundance and function [144–146, 149, 150, 153]. These regulatory axes represent potential intervention points to stabilize c-FLIP when apoptosis is maladaptive or to promote its degradation when excessive survival drives pathology.

At the immunological level, alterations in c-FLIP disrupt immune homeostasis. Overexpression impedes AICD in T cells, allowing autoreactive clones to persist and predisposing to autoimmunity [9, 189, 199, 423], whereas c-FLIP deficiency in Tregs leads to uncontrolled inflammation and lethal autoimmunity [22, 217]. In myeloid cells, c-FLIP fine-tunes inflammatory responses and cell survival; its dysregulation contributes to chronic inflammation and tumor-promoting immunosuppression by activating NF-κB and JAK-STAT3 signaling and upregulating PD-L1 [54]. These findings highlight c-FLIP as both a stabilizer of immune tolerance and a facilitator of immune escape, depending on its expression and cellular context. The role of c-FLIP in the pathogenesis of specific immune-related diseases, including asthma, IBDs, MS, and many others, has been clearly established through genetic approaches in various mouse models and then confirmed in human patients. In some cases, including Sjögren’s syndrome, the role of the protein has been hypothesized only based on its modulation in preclinical models and relatively limited human samples [326, 424–426]

Despite substantial progress, key questions persist. The isoform-specific contributions of c-FLIP_L_ and c-FLIP_S_ to apoptosis and necroptosis, their interaction hierarchies with caspase-8, and the evolutionary selection for differential expression across tumor types remain incompletely defined. Translational progress is further hindered by interspecies differences, notably the absence of c-FLIP_S_ in mouse models. To overcome these limitations, emerging platforms, including spatial multi-omics, single-cell transcriptomics, organoid systems, and 3D bioprinting, are increasingly used to capture the complexity of human disease and enable the development of more predictive preclinical models. These innovative strategies aim to enhance the clinical application of c-FLIP-targeted therapies, thereby improving the efficacy of conventional treatments and facilitating the development of novel therapies for rare diseases. In this context, downregulation of c-FLIP_L_ in melanoma has been shown to augment T cell antitumor responses, thereby enhancing the effectiveness of PD-1 blockade therapy [427].

While the therapeutic potential of targeting c-FLIP is undeniable, it must be weighed against the risks of its broad involvement in key physiological processes [428, 429]. PROteolysis TArgeting Chimeras (PROTACs) represent a rational approach to selectively degrade overexpressed c-FLIP in tumor cells, thereby restoring apoptotic sensitivity and complementing immune checkpoint blockade [430]. Combining PROTACs with nanomaterial-based delivery systems may further enhance pharmacologic precision, minimizing off-target effects and improving tumor selectivity. Conversely, therapeutic upregulation of c-FLIP through in vivo delivery of *CFLAR* mRNA encapsulated in lipid nanoparticles could promote the differentiation of tolerogenic APCs, representing a potential strategy for treating autoimmune diseases. A similar concept has been validated using PD-L1 mRNA nanotherapy, which induced tolerogenic APCs and prevented disease progression in murine models of rheumatoid arthritis and ulcerative colitis [431].

Collectively, these findings position c-FLIP as a master regulator at the intersection of apoptosis, necroptosis, and inflammation. Its ability to integrate death receptor signaling with immune and metabolic networks underscores its significance in maintaining tissue homeostasis and shaping disease outcomes. Future therapeutic strategies must leverage the context-dependent biology of c-FLIP, employing precise temporal and spatial modulation to either preserve cell survival where protective or induce cell death where resistance drives pathology. Harnessing the full biology of the highly conserved protein c-FLIP, which regulates cell survival and function, may unlock existing and novel treatment approaches in precision medicine, offering avenues to enhance cancer immunotherapy and mitigate autoimmune diseases.
